# Wiskott-Aldrich syndrome protein interacts and inhibits diacylglycerol kinase alpha promoting IL-2 induction

**DOI:** 10.3389/fimmu.2023.1043603

**Published:** 2023-04-17

**Authors:** Suresh Velnati, Sara Centonze, Giulia Rossino, Beatrice Purghè, Annamaria Antona, Luisa Racca, Sabrina Mula, Elisa Ruffo, Valeria Malacarne, Mario Malerba, Marcello Manfredi, Andrea Graziani, Gianluca Baldanzi

**Affiliations:** ^1^ Department of Translational Medicine, Universitàdel Piemonte Orientale, Novara, Italy; ^2^ Center for Translational Research on Allergic and Autoimmune Diseases (CAAD), Università del Piemonte Orientale, Novara, Italy; ^3^ Department of Heath Sciences, Università del Piemonte Orientale, Novara, Italy; ^4^ Department of Molecular Biotechnology and Health Sciences, Molecular Biotechnology Center (MBC), University of Turin, Turin, Italy; ^5^ Department of Surgery and Immunology, University of Pittsburgh, Pittsburgh, PA, United States; ^6^ Respiratory Unit, Sant’Andrea Hospital, Vercelli, Italy

**Keywords:** DGK diacylglycerol kinase, WAS (Wiskott-Aldrich syndrome), T cell receptor signalling, SLAM-associated protein (SAP), restimulation-induced cell death, X-linked lymphoproliferative disease (XLP)

## Abstract

**Background:**

Phosphorylation of diacylglycerol by diacylglycerol-kinases represents a major inhibitory event constraining T cell activation upon antigen engagement. Efficient TCR signalling requires the inhibition of the alpha isoform of diacylglycerol kinase, DGKα, by an unidentified signalling pathway triggered by the protein adaptor SAP. We previously demonstrated that, in SAP absence, excessive DGKα activity makes the T cells resistant to restimulation-induced cell death (RICD), an apoptotic program counteracting excessive T cell clonal expansion.

**Results:**

Herein, we report that the Wiskott-Aldrich syndrome protein (WASp) inhibits DGKα through a specific interaction of the DGKα recoverin homology domain with the WH1 domain of WASp. Indeed, WASp is necessary and sufficient for DGKα inhibition, and this WASp function is independent of ARP2/3 activity. The adaptor protein NCK-1 and the small G protein CDC42 connect WASp-mediated DGKα inhibition to SAP and the TCR signalosome. In primary human T cells, this new signalling pathway is necessary for a full response in terms of IL-2 production, while minimally affecting TCR signalling and restimulation-induced cell death. Conversely, in T cells made resistant to RICD by SAP silencing, the enhanced DAG signalling due to DGKα inhibition is sufficient to restore apoptosis sensitivity.

**Conclusion:**

We discover a novel signalling pathway where, upon strong TCR activation, the complex between WASp and DGKα blocks DGKα activity, allowing a full cytokine response.

## 1 Introduction

Diacylglycerol kinases (DGK) tune lipid signalling by metabolizing diacylglycerol (DAG) to phosphatidic acid. Two isoforms, namely DGKα and DGKζ, play a major role as negative regulators of DAG-mediated TCR signalling. This signalling includes the Ras guanyl nucleotide-releasing protein 1 (RasGRP1)/Ras/mitogen-activated protein kinases (MAPKs) pathway, the activator protein 1 (AP1) transcription factor activity, and the interleukin 2 (IL-2) induction (1). The activity of both isoforms promotes T cell anergy, a tolerance condition characterized by the uncoupling of ligand-induced TCR activation from downstream signalling, while their inhibition potentiates T cell activation and IL-2 synthesis (2, 3). In addition, DGKα and DGKζ contribute to tumour cell immune escape, and their inhibition rescues defective anti-tumour killing activity in tumour-specific exhausted T cells (4, 5). Quantitative studies indicate that DGKζ is responsible for the metabolism of most of the DAG generated by phosphatidylinositol 4,5-bisphosphate (PIP_2_) hydrolysis and controls the activation of DAG-effectors such as protein kinase C theta (PKCθ) and RasGRP1 (6, 7). Conversely, DGKα activity is rapidly recruited in a phosphatidylinositol (3–5)-trisphosphate (PIP_3_)-dependent manner to phosphorylate a minor pool of DAG at the periphery of the immune synapse, playing a key function in shaping the immune synapse and cell polarization (8).

Consistent with their role as negative regulators, the expression of both DGKα and DGKζ is downregulated 3-5-fold upon antigen-induced T cell differentiation into effector cells through the PIP_3_ signalling pathways (9, 10). The simultaneous triggering of CD3 and CD28 also causes a specific and rapid downmodulation of DGKα enzymatic activity. This downmodulation requires PLC activity and the small SH2-containing adaptor protein SAP (SLAM-associated protein) (11). Loss of function mutations of the SAP encoding gene SH2D1A causes X-linked lymphoproliferative disease type 1 (XLP-1), a primary immunodeficiency characterized by multiple defects in the T and NK cell compartments leading to EBV-triggered hemophagocytic lymphohistiocytosis (HLH), hypogammaglobulinemia, and predisposition to lymphomas (12). In T cells from XLP-1 patients, SAP absence abolishes the costimulatory function of the signalling lymphocytic activation molecule (SLAM) family of membrane receptors and facilitates the recruitment of the inhibitory tyrosine phosphatases Src homology phosphatase 1 and 2 (SHP-1 and SHP-2). This reduces TCR signalling strength to the nuclear factor of activated T-cells (NF-AT) and the induction of interleukins such as IL-2 and IL-10 along with proapoptotic mediators like FAS ligand, Bcl-2 Interacting Mediator of cell death (BIM), and nuclear receptor 4A1 (NUR77). The typical predisposition to HLH of XLP-1 patients is due to two factors: i) decreased signalling strength and modified assembly of the TCR signalosome, which leads to lymphocyte accumulation due to defective restimulation-induced cell death (RICD), and ii) altered immune synapse morphology which decreases cytotoxic T cells (CTL) efficiency in removing Epstein-Barr virus (EBV) infected B cells (13). Those functional defects in T cells are in part due to excessive DGKα activity that, in the absence of SAP, blunts T cell signalling. Although the molecular mechanisms connecting SAP and DGKα are uncharacterized, inhibition or silencing of DGKα in SAP-deficient T cells rescues immune synapse morphology and DAG signalling strength along the Ras/MAPK, PKCθ, and NF-AT signalling pathways. Moreover, DGKα inhibitors are also effective in rescuing some aspects of the immunopathology of XLP-1 murine models, indicating a crucial role of DGKα in the XLP-1 pathology onset (14). Interestingly, while knockdown of DGKα in SAP deficient cells rescues the signalling machinery necessary for RICD and IL-2 expression, other crucial effectors such as FASL remain downregulated, indicating the existence of diverging SAP-dependent signal pathways (14, 15). Indeed, SAP participates in multiple crucial interactions in T cells such as the recruitment of Fyn and Lck tyrosine kinases to SLAM receptors (16, 17), the recruitment of other SH3-containing adaptors such as NCK adaptor protein 1 (NCK-1) (18), and the activation of Rac/cell division control protein 42 (CDC42) small G proteins through the pak interacting exchange factor (βPIX) which in turn drives NF-AT activation (19).

To better understand the signalling mechanism by which SAP negatively regulates DGKα activity, we focused on the Wiskott–Aldrich syndrome protein (WASp) as this protein is regulated by NCK-1 binding to the WASp proline-rich region mediating its localization, and CDC42 binding to CRIB domain of WASp promoting its activation (20, 21). Moreover, it was already reported that lymphocytes from WASp-deficient mice have defective RICD, reinforcing the notion of the existence of a SAP-WASp signalling pathway (22). WASp belongs to the nucleation-promoting factors (NPFs) family and by activating the actin-related protein 2/3 (ARP2/3) complex, it facilitates actin polymerization at the T cell immune synapse (23, 24) but also NF-AT nuclear translocation for IL-2 induction (25). Loss of function mutations in WASp gives rise to the Wiskott–Aldrich syndrome, which is characterized by bleeding, thrombocytopenia, eczema, frequent infections, and susceptibility to the development of autoimmune diseases and lymphomas (26). Thus, the signalling connections and some similarities between those two primary immunodeficiencies (27) prompted us to explore the role of WASp and CDC42 in regulating DGKα activity and DAG-dependent T cell activation.

## 2 Materials and methods

All the chemical reagents used in this study are listed in **Table 1**.

**Table 1:** Detailed list of lab reagents used in the present work.

Antibodies				
Antibody		Clone	Cat. number	Provider
Anti-human CD3		UCHT1	MA1-80537	Invitrogen
Anti-human CD3		OKT3	40-0037-U500	Tonbo biosciences
Anti-human CD28		CD28.2	16-0289-85	Invitrogen
Anti-DGKα		–	11547-1-AP	Proteintech
Anti-DGKα		–	AB64845	Abcam
Anti-Phospho-p44/42 MAPK (Erk1/2)		–	9101	Cell Signalling Technology
Anti-p38 MAPK		–	9212S	Cell Signalling Technology
Anti-WASp		D9C8	4271	Cell Signalling Technology
Anti-WASp		–	4860	Cell Signalling Technology
Anti-WASp		–	Sc-13139	Santa Cruz Biotechnology
Anti-CDC42		–	Sc-87	Santa Cruz Biotechnology
Anti NCK-1		5B7	12778	Cell Signalling Technology
Anti-Vinculin		hVIN-1	V9264	Sigma-Aldrich
Anti-b-actin		8H10D10	3700	Cell Signalling Technology
Anti- c-myc		9E10	MA1-980-1MG	Invitrogen
Anti- c-myc- AC		9E10	sc-40 AC	Santa Cruz Biotechnology
Anti-ECS (DDDDK) HRP conjugated		–	A190-101P	Bethyl laboratories
Anti-FLAG HRP conjugated		–	HRP-66008	Proteintech
Anti-FLAG		M2	A2220	Sigma-Aldrich
Anti-DYKDDDDK tag		FG4R	MA1 91878	Life technologies
Anti-DYKDDDDK tag HRP-Conjugated		FG4R-HRP	MA1 91878-HRP	Invitrogen
Anti-GST		Z-5	sc-459	Santa Cruz Biotechnology
Anti-human IL-2 (APC)		MQ1-17H12	500310	Biolegend
Chemical Compounds				
Name		Function	Cat. number	Provider
rhIL-2		IL-2	200-02	Preprotech
GST- WAS (Human) Recombinant protein (P01)		WASp	H00007454-P01	Abnova
CK-666		ARP2/3 Inhibitor	SML0006-5MG	Sigma Aldrich
AMB639752		DGKα inhibitor		Ambinter
Jasplakinolide		Actin stabilizer	AG-CN2-0037-C100	Vinci Biochem
Brefeldin A (BFA)		Protein transport inhibitor	B6542	Sigma Aldrich
siRNAs (Where not specified, siRNAs are from Thermo Fisher Scientific (Life Technologies))				
Target	Technology	siRNA ID & Cat. #	Sequence	
SAP	Stealth RNAi™ siRNA	siRNA ID: Custom	Forward	UGUACUGCCUAUGUGUGCUGUAUCA
		Cat. # 10620312	Reverse	UGAUACAGCACACAUAGGCAGUACA
WASp	Silencer™ Select Pre-Designed siRNA	siRNA ID: s14836	Forward	UGAACAACCUCGACCCAGAtt
		Cat. # 4392420	Reverse	ACUGGGUCGAGGUUGUUCAcg
CDC42	Silencer™ Select Pre-Designed siRNA	siRNA ID: s2765	Forward	UGGUGCUGUUGGUAAAACAtt
		Cat. # 4392420	Reverse	UGUUUUACCAACAGCACCAtc
NCK-1	Silencer™ Select Pre-Designed siRNA	siRNA ID: s9311	Forward	CCUCAUUCGUGAUAGUGAAtt
		Cat. # 4392421	Reverse	UUCACUAUCACGAAUGAGGaa
DGKα	Stealth RNAi™ siRNA	siRNA ID: Custom	Forward	CGAGGAUGGCGAGAUGGCUAAAUAU
		Cat. # 10620312	Reverse	AUAUUUAGCCAUCUCGCCAUCCUCG
SYBR green Primers (where not specified, SYBR green primers are from Sigma-Aldrich (Merck)).				
Gene		Code	Primer	Sequence
DGKA		FH1_DGKA	Forward	AATGTTCCCAGACACCTAAG
		RH1_DGKA	Reverse	AGTAGCAGGAAACATCATTG
SH2D1A		FH1_SH2D1A	Forward	AAGGGATAAGAGAAGATCCTG
		RH1_SH2D1A	Reverse	CATTACAGGACTACAATGGC
WAS		FH1_WAS	Forward	TACTCACAGCTTGTCTACTC
		RH1 WAS	Reverse	TTTTGTATCTTCTCCTGCAC
CDC42		FH1 CDC42	Forward	GAACAAACAGAAGCCTATCAC
		RH1 CDC42	Reverse	TTTAGGCCTTTCTGTGTAAG
NCK-1		FH1_NCK1	Forward	CCAAGTATATTGTGTCTGCC
		RH1_NCK1	Reverse	CTATGTCTCATGTGTCTTGC
GAPDH		FH1_GAPDH	Forward	TTGAGCACAGGGTACTTTA
		RH1_GAPDH	Reverse	ACAGTTGCCATGTAGACC
TaqMan probes (Where not specified, TaqMan probes are from Thermo Fisher Scientific (Life Technologies)).				
Gene			Cat. number	
IL-2			Hs00174114_m1	
GAPDH			Hs02758991_g1	
NR4A1			Hs00374226_m1	
FASLG			Hs00181225_m1	
FOS			Hs04194186_s1	
IFNγ			Hs00989291_m1	
Buffers				
Buffer	Composition			
Lysis buffer	25 mM HEPES, 150 mM NaCl, 5 mM EDTA, 1 mM EGTA, 1% NP40, 10% glycerol, (supplemented with 1 mM orthovanadate along with protease inhibitors before use), pH 8.0			
LiCl_2_ buffer	25 mM Tris, 0.5 M LiCl, pH 8.0			
TNE buffer	25 mM Tris, 150 mM NaCl, 1 mM EDTA, pH 8.0			
Homogenization buffer	25 mM Hepes (pH 8), 20% glycerol, 135 mM NaCl, 5 mM ethylenediaminetetraacetic acid (EDTA), 1 mM ethylene glycol-bis (beta-aminoethyl ether)-N,N,N′,N′-tetra acetic acid (EGTA), 1 mM sodium orthovanadate and protease inhibitor cocktail			
Elution buffer	100 mM Tris HCl, 10 mM NaCl, supplemented with fresh 2 mM DTT, and glutathione 10 mM, pH 8.0			
Laemmli sample buffer	187.4 mM Tris HCl, 30% glycerol, 6% SDS, bromophenol blue in sufficient quantity, (supplemented with DTT 150 mM before use), pH 6.8			
TBS-T	50 mM Tris, 120 mM NaCl, 0.01% TWEEN20 detergent			
PBS	137 mM NaCl, 2.7 mM KCl, 4.3 mM Na_2_HPO_4_, and 1.5 mM KH_2_PO_4_, pH 7.4			
Plasmids and mutants				
Plasmid		Reference		Cat. number
myc-DGKα		Cutrupi et al., 2000		
GST-DGKα		Baldanzi et al., 2008		
FLAG-WASp				47432
myc-FLAG-N-WASp				RC207967
myc-DGKα L360*		Baldanzi et al., 2008		
To create mutant variants of the above-mentioned plasmids we used Phusion™ Site-Directed Mutagenesis Kit (Thermo Fisher Scientific) according to the manufacturer’s instructions. Mutagenesis was performed using the following primers:				
Mutant	Sequence			
DGKα E86*	Forward	TCACTGCTTAAATTAGACAAATGTGACAAA		
	Reverse	CCAGTCTCAAAGGATTGAAACAGTGC		
WASp K232*	Forward	CTCAGGGAAGAAGTAGATCAGCAAAG		
	Reverse	CGTTTCTTATCAGCTGGGCTAGG		
WASp Q297*	Forward	TTCATTGAGGACTAGGGTGGGCTGG		
	Reverse	GTCGTAGATAAGTTTAGAGGTCTCGGCG		
shRNA plasmids				
Target	Vector plasmid		Cat. number	Provider
WASp	MISSIONpLKO.1-puro Empty Vector Plasmid DNA		TRCN0000029819	Sigma-Aldrich
NT (Non targeting)	MISSIONpLKO.1-puro Empty Vector Plasmid DNA		SHC002	Sigma-Aldrich

### 2.1 Cell culturing

Human embryonic kidney 293T cells were cultured in 100 mm plates using DMEM with 10% FBS (Lonza) and 1% penicillin/streptomycin (Life Technologies).

Jurkat cells were cultured in RPMI-GlutaMAX (Life Technologies) with 10% FBS (Lonza) and 1% penicillin/streptomycin (Life Technologies).

Peripheral blood mononuclear cells (PBMCs) were isolated from healthy anonymous human buffy coats (provided by the Transfusion Service of Ospedale Maggiore della Carità, Novara, Italy). PBMCs were isolated by Ficoll-Paque PLUS (GE Healthcare) density gradient centrifugation, washed, and resuspended at 2 x 10^6^ cells/ml in RPMI-GlutaMAX containing 10% heat-inactivated FBS (Lonza), 2 mM glutamine, and 100 U/ml of penicillin and streptomycin (Life Technologies). T cells were activated with 1 μg/ml anti-CD3 (clone UCHT1) and anti-CD28 (clone CD28.2) antibodies for 72 hours. Activated T cells were then washed and cultured in the complete medium along with 100 IU/ml rhIL-2 (PeproTech) at 1-2 x 10^6^ cells/ml for ≥ 7 days by changing media every 2–3 days.

### 2.2 Generation of Jurkat WASp shRNA and the Jurkat control shRNA

To generate stable WASp-silenced Jurkat cells we used MISSIONpLKO.1-puro Empty Vector Plasmid DNA (Sigma-Aldrich) harbouring either the sequence targeting human WASp (WASp *shRNA*) or a non-targeting short hairpin RNA (*shRNA*). Jurkat cells were plated and transduced with lentiviral particles in the presence of polybrene (6 µg/ml: Sigma-Aldrich, Saint Louis, MO, USA). The spinoculation protocol was performed as follows: the plate was centrifuged for 30 minutes at 32^°^C, 800 g. Subsequently, the medium containing viral particles was removed, and cells were resuspended in a fresh medium and incubated for three days (37^°^C, 5% CO_2_). Lastly, 1 µg/ml puromycin (Sigma-Aldrich, Saint Louis, MO, USA) was added for the selection.

### 2.3 Jurkat cell stimulations and Immunoprecipitation for activity assays

Jurkat cells (Control *shRNA* and WASp *shRNA*) were cultured at a concentration of 1 x 10^6^ cells/ml for 3 continuous weeks. 3 x 10^7^ cells for each condition were resuspended in RPMI 1640 (preheated at 37^°^C) and/or pre-treated with DMSO or ARP2/3 inhibitor and/or DGKα inhibitor for 30 minutes. After pre-treatment, cells were stimulated using anti-CD3 (UCHT1 clone) at a concentration of 10 μg/ml for 15 minutes and cells were lysed in 1 ml of lysis buffer. An aliquot of 50 µl cell lysates was retained for western blot analysis and the remaining cells were subjected to immunoprecipitation using 5 µg/ml of anti-DGKα antibodies (Abcam-AB64845) for 4 hours using protein G agarose beads in continuous rotation at 4^°^C. After immunoprecipitation, beads with DGKα antibody were washed twice with lysis buffer followed by 2 washes in LiCl_2_ and 2 in TNE buffer. Following this, the DGKα immunoprecipitates were subjected to DGK assay to measure DGK activity.

### 2.4 Preparation of 293T homogenates for activity assays

293T cells were transiently transfected with indicated plasmid DNA using Lipofectamine 3000, Invitrogen (Carlsbad, CA) in 10 cm^2^ plates. 48 hours post-transfection, cells were harvested and homogenized by passing them 30 times through a 29G-needle using 500 μl of homogenization buffer for each dish and immediately stored in aliquots at -80^°^C. Cells transfected with GFP were used as controls.

### 2.5 DGK activity assay with ATPγP32

The same procedure was followed as reported previously in (11). DGK activity was assayed by measuring initial velocities (5 minutes at 27^°^C) in the presence of saturating substrate concentration. Reaction conditions were 0.9 mg/ml 1,2-dioleoyl-sn-glycerol, 5 mM ATP, 0.01 mCi/ml [c^32^P]-ATP (for homogenates) or 0.04 mCi/ml [c^32^P]-ATP (for immunoprecipitates), 1 mM sodium orthovanadate, 10 mM MgCl_2_, and 1.2 mM EGTA in 7.5 mM HEPES pH 8. The reaction mixture was assembled by mixing enzyme (25 µl of either cell homogenates or immunoprecipitated endogenous DGKα), 5X ATP solution, and 3X DAG solution. The reaction was terminated after 5 minutes by adding 200 µl of freshly prepared 1 M HCl and lipid was extracted by adding 200 µl of CH_3_OH : CHCl_3_ 1:1 solution and vortexing for 1 minute. The two phases were separated by centrifugation (12,000 RCF for 2 minutes). Either 25 µl (for homogenates) or 50 µl (for DGKα immunoprecipitates) of the lower organic phase was spotted in small drops on silica TLC plates. TLC was run 10 cm and dried before radioactive signals were detected by GS-250 molecular imager and were quantified by either quantity one 4.01 or image lab 6.0 (Bio-Rad, Hercules, CA) software assuring the absence of saturated spots.

### 2.6 GST-DGKα protein purification

293T cells were transfected with GST-DGKα construct or GFP construct (Lonza) as a control, using lipofectamine 3000 reagent (Thermo Fisher Scientific) according to the manufacturer’s instruction. After 48 hours, cells were lysed in 1 ml of lysis buffer supplemented with 2 mM dithiothreitol and centrifuged at 12,500 rpm at 4°C for 15 minutes. Afterwards, the lysates were incubated with 150 µl of glutathione-agarose beads (GE Healthcare) in gentle agitation (4 hours at 4°C). After three washes in lysis buffer and three in PBS, the pulled-down proteins were eluted with 90 µl GST elution buffer (10 minutes at 37°C). Protein purification was verified by SDS-PAGE stained with EZBlue™ Gel Staining Reagent (Merck). Purified protein quantification was done by densitometry using BSA standards and the ChemiDocTM Imaging System (Bio-rad).

### 2.7 Luciferase based DGKα activity assay

Purified GST-DGKα activity was measured in the presence and absence of an excess of commercial GST-WASp protein (Abnova). We used the DGKA Kinase Enzyme System (Promega) and ADP-Glo Kinase Assay kit (Promega) following the manufacturer’s instructions. The assay was performed in a total volume of 5 μl (white 384-well microplate) comprising 100μM dilauroylglycerol, 50 µM DTT, 100 µM ATP and, where indicated, 2mM CaCl_2_. After 90 minutes of incubation, the ADP Glo reagent was added (5 µl/well; 40 minutes) followed by Kinase Detection Reagent (10 µl/well; 40 minutes). The luminescence was measured using Tecan Spark 10 M Multimode Plate Reader. E. coli DGKA (Merk) was used as a positive control, while mock purifications from GFP-transfected cells and no enzyme samples were used as background signals. The data were analysed as:

DGKα activity = (sample luminescence – no enzyme)/(GST-DGKα activity – no enzyme) *100

### 2.8 Lipidomic analysis

Jurkat cells were starved overnight in RPMI 0% FBS at a concentration of 2 x 10^6^ cells/ml. The following morning, cells were washed in PBS and stimulated using anti-CD3 (OKT3 clone) + CD28 (CD28.2) antibodies at a concentration of 1 µg/ml for 15 minutes at 37^°^C. Post-stimulation, cells were washed four times in ice-cold PBS by centrifugation (700 g for 5 minutes) and, after the last wash, the pellet was dried and subjected to lipidomic analysis.

Cells were extracted using 1 ml of 75:15 IPA/H_2_O solution, after the addition of 100 μl of CH_3_OH 5% deuterated standard (Splash Lipidomix^®^). Then the samples were vortexed for 30 seconds, sonicated for 2 minutes, vortexed again for 30 seconds, and then were incubated for 30 minutes at 4^°^C, under gentle, constant shaking. Subsequently, samples were rested on ice for additional 30 minutes. To remove debris and other impurities, the samples were centrifuged for 10 min at 3500 g at 4°C. 1 ml of supernatant was collected and dried using a SpeedVac centrifuge (Labogene). The dried samples were reconstituted in 100 μl of CH_3_OH containing the internal standard CUDA (12.5 ng/ml).

For the analysis of the reconstituted lipids, a UHPLC Vanquish system (Thermo Scientific, Rodano, Italy) coupled with an Orbitrap Q-Exactive Plus (Thermo Scientific) was used.

Mass spectrometry analysis was performed in positive ion mode. The source voltage was maintained at 3.5 kV. The capillary temperature, sheath gas flow, and auxiliary gas flow were set at 320°C, 40 arb, and 3 arb respectively. S-lens was settled at 50 rf. Data were collected in a data-dependent (ddMS2) top 10 scan mode. Survey full-scan MS spectra (mass range m/z 80 to 1200) were acquired with resolution R = 70,000 and AGC target 1e6. MS/MS fragmentation was performed using high-energy c-trap dissociation (HCD) with resolution R = 17,500 and AGC target 1e5. The stepped normalized collision energy (NCE) was set to 15, 30, and 45 respectively. The injection volume was 3 µl. Lockmass and regular inter-run calibrations were used for accurate mass-based analysis. An exclusion list for background ions was generated by analysing a procedural blank sample. The acquired raw data were processed using MSDIAL software (Yokohama City, Kanagawa, Japan), version 4.24.

A QC-based regression model using linear weighted scatter plot smoothing (LOWESS) was used to adjust real sample signals according to the analytical order. Results were then reported as normalized peak intensity.

### 2.9 Proximity ligation assay

Isolated human PBLs (0.15 x 10^6^) were seeded in poly-L-lysine coated glasses in a 24-well plate and treated with the indicated stimuli. The cells were washed with ice-cold PBS, fixed in 3% paraformaldehyde/4% sucrose pH 7.4 for 10 minutes, permeabilized in ice-cold HEPES-Triton X-100 buffer (HEPES pH 7.4 20 mM, sucrose 300 mM, NaCl 50 mM, MgCl_2_ 3 mM, Triton X-100 0.5%) for 5 minutes, washed twice with PBS, and processed according to the PLA (Duolink, Merck, cat. DUO92101) manufacturer’s protocol. The samples were imaged with TCS SP5 confocal microscope (Leica Microsystem) as z stack of 1 μm thickness to cover the entire volume of the cells with a Plan Apo 63 X (NA 1.4) oil objective. PLA dots were quantified manually on maximum projection images obtained with Image J software (NIH).

### 2.10 Complex formation assays

293T cells were plated in 100 mm diameter Petri dishes and allowed to reach 90% confluence. Transfections were performed with the indicated DNA plasmids using Lipofectamine 3000 reagent according to the manufacturer’s instructions (Invitrogen by Thermo Fisher). After 48 hours, cells were washed in ice-cold PBS and lysed in 1 ml of lysis buffer. Subsequently, lysates were incubated with 5 µg/ml anti-FLAG antibody (Sigma-Aldrich) or with 5 µg/ml anti-myc antibody agarose conjugated (Santa Cruz Biotechnology) and 20 µl Protein G Sepharose beads Fast Flow (Sigma) at 4°C. An hour after incubation, beads were washed in lysis buffer six times and boiled in Laemmli Sample Buffer for 5 minutes at 95°C. Thus, the obtained immunoprecipitates were loaded on SDS-PAGE and analysed by western blotting using ChemiDoc™ Imaging System (Bio-Rad).

### 2.11 Restimulation-induced cell death assays in human peripheral T cells

Activated human PBLs were transfected with 200 pmol of siRNA oligonucleotides specific for the target protein or a non-specific Stealth RNAi Negative Control Duplexes (12935–300) (Stealth Select siRNA; Life Technologies). Transient transfections were performed using Amaxa nucleofector kits for human T cells (Lonza) and either the Amaxa Nucleofector 4D systems (programs E0-115) or the Amaxa Nucleofector II system (program “primary lymphocyte T-020”). Cells were cultured in IL-2 (100 IU/ml) for 4 days to allow target gene knockdown. Knockdown efficiency was periodically evaluated either by Western blotting or by rt-PCR.

To test restimulation-induced cell death (RICD), activated T cells (10^5^ cells/well in 200 µl) were plated in either triplicates or quadruplicates in a 96-well round-bottom plate and treated with increasing concentrations of anti-CD3 (clone OKT3) of 0, 10, and 100 ng/ml in RPMI-GlutaMAX supplemented with 100 IU/ml rhIL-2 for 24 hours. In these assays, DGK inhibitor AMB639752 (10 μM) was added 30 minutes before the restimulation with OKT3. 24 hours after treatment, cells were stained with 20 ng/ml propidium iodide and collected for a constant volume of 150 µl per sample on Attune Nxt Flow Cytometer (Thermo Fisher Scientific). Cell death is expressed as % cell loss and calculated as

% Cell loss = (1-(number of viable cells in sample/number of viable cells in control) x 100)

Results were expressed as mean ± standard error mean (SEM). We always compare controls and WASp silenced lymphocytes from the same donors as there is a large individual variability in RICD sensitivity.

### 2.12 Gene expression assays

Activated human PBLs were transfected as described above and, 4 days after transfection, cells were collected, washed, and seeded at the concentration of 1.5 x 10^6^ cells in 1 ml of RPMI supplemented with 10% FBS and 100 IU/ml rhIL-2. Subsequently, cells were pre-treated for 30 minutes with the indicated inhibitors or DMSO and stimulated with OKT3 + CD28.2 at the concentration of 1 µg/ml. After 4 hours, cells were collected, washed twice with ice-cold PBS, and subjected to RNA extraction using RNeasy Plus mini kit from Qiagen or Maxwell RSC simply RNA extraction kit (AS1390, Promega Italia), following manufacturer instructions. The RNA concentration and purity were estimated by a spectrophotometric method using Nanodrop 2000c (Thermo Scientific). The RNA was retrotranscribed with LunaScript RT Supermix kit (NEB) or High-Capacity cDNA Reverse Transcription Kits (Applied Biosystems). The resulting cDNA was quantified by real-time PCR (Luna Universal qPCR Master Mix by NEB or ABI7900 by Applied Biosystems) in either 96 or 384 well plates using GUSB or GAPDH as normalizers.

WASp shRNA transduced Jurkat cells were collected periodically along with control shRNA transduced Jurkat cells to check the efficiency of WASp silencing.

### 2.13 IL-2 protein detection in PBLs

Activated primary T cells were nucleofected with siRNAs against WASp, DGKα, or both. After 4 days, 0.6 x 10^6^ cells were seeded in 400 µl RPMI 10% FBS and stimulated or not on plate-bound anti-CD3 + anti-CD28 (clone UCHT1, 10 µg/ml and clone CD28.2 5 µg/ml, respectively) for 6 hours. After 1 hour of incubation, the protein transport inhibitor brefeldin A (BFA) 5 µg/ml (Merck) was added for the remaining 5 hours. Then, cells were fixed and permeabilized with BD Cytofix/Cytoperm kit according to the manufacture’s protocol, stained with APC anti-human IL-2 Antibody (clone MQ1-17H12, Biolegend) for 30 minutes on ice and assayed through BD FACSCelesta with BD FACSDiva software. Data are analysed with FCS Express 7.

### 2.14 Western blot analysis

To verify the protein expression in immunoprecipitated Jurkat cells or 293T cells, equal amounts of protein lysates or homogenates were analysed by western blotting as follows. Proteins were separated on SDS–PAGE gels and subsequently transferred to a polyvinylidene fluoride (PVDF) membrane. The membranes were then blocked by incubating with 3% (w/v) dried BSA for 1 hour at room temperature and subjected to overnight incubation with respective primary antibodies at 4°C with gentle agitation. The following day, membranes were washed 4 times in TBS-T buffer at 15 minute time intervals. They were then incubated with alkaline HRP-conjugated rabbit or mouse secondary antibody (1:5000, PerkinElmer, USA) and diluted in TBS-T for 1 hour at room temperature with gentle agitation. After four additional 15 minute washes with TBS-T, the membranes were developed using the Western Chemiluminescence Substrate (PerkinElmer, USA) using the ChemiDoc™ Imaging System (Bio-rad).

### 2.15 Statistical analysis

Evaluation of all the *in vitro* assays across multiple treatments, DGK activity assays, homogenates activity assays, RICD assays, and gene expression assays were analysed by using either one-way ANOVA or two-way ANOVA with multiple comparisons’ correction using either GraphPad PRISM 9.0 or 9.4 software. Single treatments of lipidomic data were analysed with Student T-test using GraphPad PRISM software. Error bars are described in Figure legends as ± SEM or ± SD where appropriate. A single, double, triple, and four asterisks denote their significance of a p-value ≤ 0.05, ≤ 0.01, ≤ 0.001, and ≤ 0.0001 respectively in all experiments.

## 3 Results

### 3.1 WASp is necessary for DGKα inhibition upon TCR activation

We have previously reported that T cell co-stimulation with OKT3 and CD28.2 results in a SAP-mediated inhibition of the DGKα activity (11). Similarly to SAP, WASp is an adaptor protein tuning the TCR signalling which leads to cytokine expression and RICD (22, 24). Thus, we investigated whether WASp is involved in the signalling pathway triggered by TCR which leads to DGKα inhibition. As we observed that the sole triggering of the TCR with the strong agonist antibody UCHT1 is sufficient for DGKα inhibition, we used this condition in the following experiments.

**Figure 1:**
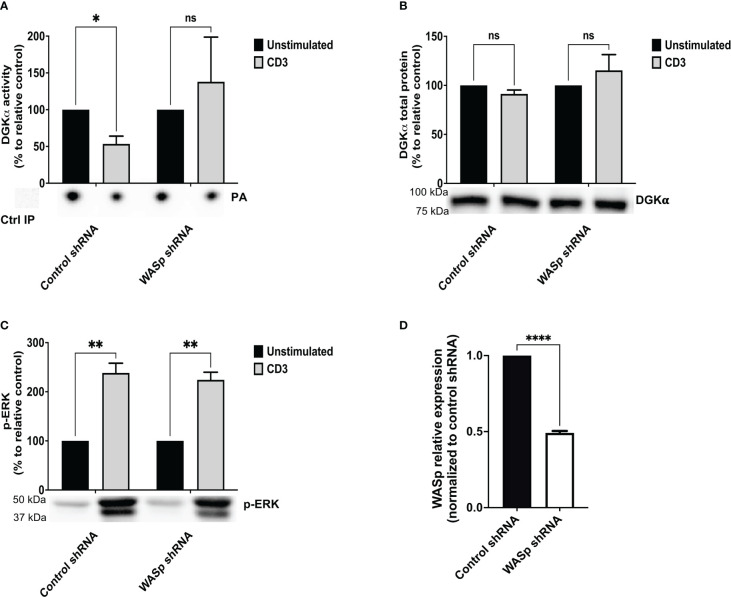
WASp is required for DGKα inhibition upon CD3 stimulation Control shRNA and WASp shRNA Jurkat cells (3 x 10^7^) were stimulated with CD3 agonist (UCHT1 10 μg/ml for 15 minutes). Post-stimulation, cells were lysed and: **(A)** immunoprecipitated with anti-DGKα antibody followed by DGK activity assays; the lower panel is a representative assay and upper panel is the mean ± SEM of 4 experiments normalized as the % of unstimulated cells. **(B)** Total cell lysates were analysed by western blotting with anti DGKα antibodies; the lower panel is a representative blot and upper panel is the mean ± SEM of 4 experiments normalized as the % of unstimulated cells. **(C)** Total cell lysates were analysed by western blotting with anti-pERK antibodies; the lower panel is a representative blot and upper panel is the mean ± SEM of 4 experiments normalized as the % of unstimulated cells. **(D)** WASp mRNA levels were measured by qRT-PCR at three different timepoints during the experiments and shown as mean ± SEM using control shRNA as reference. ns, non significant. A single, double, triple, and four asterisks denote their significance of a p-value ≤ 0.05, ≤ 0.01, ≤ 0.001 and ≤ 0.0001 respectively in all experiments.

Initially, WASp expression was downregulated in Jurkat cells by lentiviral-mediated stable expression of a WASp-specific short hairpin RNA (*shRNA).* WASp silencing was monitored regularly to verify the efficiency of WASp knockdown and we observed a decrease of mRNA of around 50%, considering control *shRNA* (non-targeting *shRNA)* transduced Jurkat cells as a suitable control (**Figure 1D**). Following 15 minutes of stimulation with CD3 agonist antibody (UCHT1 – 10 µg/ml), the enzymatic activity of immunoprecipitated DGKα was measured in Jurkat cells transduced with WASp *shRNA* or control *shRNA*. Consistently with previous results, the DGKα enzymatic activity was reduced by ∼50% upon CD3 stimulation in control *shRNA* Jurkat cells. Conversely, DGKα activity was not reduced upon stimulation in WASp *shRNA* Jurkat cells (**Figure 1A**), indicating that WASp is required for the negative regulation of DGKα activity upon TCR stimulation. In the same assays, western blot analysis confirmed that DGKα protein content does not change upon TCR triggering in both Jurkat cell lines (**Figure 1B**), indicating that this regulation is not due to changes in protein expression levels. We also verified that DGKα protein is reproducibly immunoprecipitated independently of the cell type (control shRNA versus WASp shRNA) or stimulation (UCHT1 10 µg/ml) (**Supplementary Materials 1**).

TCR induction of the MAPK pathways is partially dependent on the DAG activation of the RasGRP1 (28). To evaluate the DAG signalling in the absence of WASp, we assessed the phosphorylation status of ERK1/2 in the same samples. We observed a threefold increase in ERK1/2 phosphorylation upon UCHT1 treatment, which was not consistently influenced by WASp silencing (**Figure 1C**). Those data suggest that upon intense CD3 stimulation by UCHT1, WASp is relatively dispensable for TCR signalling on the ERK1/2 pathway. Moreover, the finding that ERK1/2 activation is normal in WASp-silenced cells despite high DGKα activity indicates that the DAG pool phosphorylated by DGKα is relatively dispensable for TCR signalling in those conditions. To directly evaluate the influence of WASp on T-cell DAG levels, we used a targeted mass spectrometry approach to quantify the DAG species. When Jurkat cells were stimulated with OKT3 (1 μg/ml) and CD28.2 (1 μg/ml) for 15 minutes, we observed a significant increase in selected DAG species (**Supplementary Materials 2** and Supplementary **Table S1**). The same species also increase in WASp silenced cells upon TCR + CD28 co-stimulation, confirming that the DAG pool regulated by DGKα under the control of WASp is quantitatively small.

**Figure 2:**
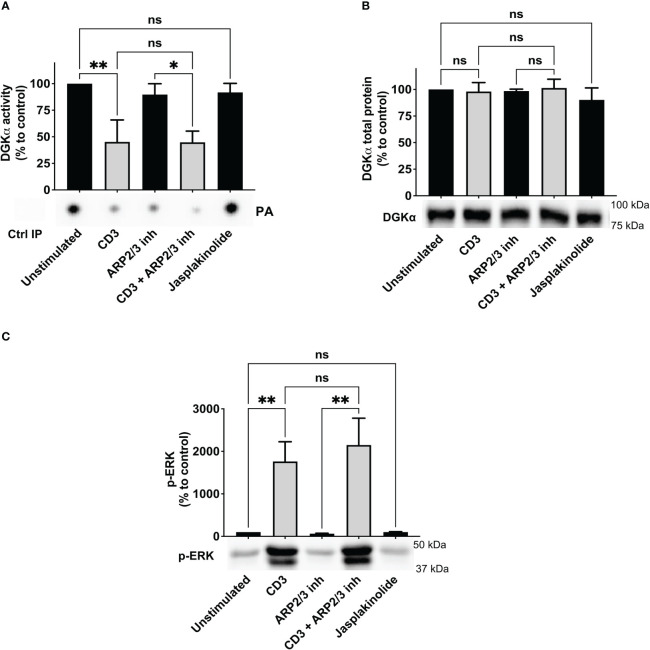
ARP2/3 has no effect on DGKα inhibition upon CD3 stimulation Jurkat cells were treated with ARP2/3 inhibitor (CK666 – 60 µM) or jasplakinolide (0.5 μg/ml) for 30 minutes and/or were stimulated with CD3 agonist (UCHT1 10 μg/ml for 15 minutes). Post-stimulation, cells were lysed and: **(A)** immunoprecipitated with anti-DGKα antibody followed by DGK activity assays; the lower panel is a representative assay and the upper panel is the mean ± SEM of at least three experiments normalized as the % of unstimulated cells. **(B)** Total cell lysates were analysed by western blotting with anti DGKα antibodies; the lower panel is a representative blot and the upper panel is the mean ± SEM of at least three experiments normalized as the % of unstimulated cells. **(C)** Total cell lysates were analysed by western blotting with anti-pERK antibodies; the lower panel is a representative blot and upper panel is the mean ± SEM of at least three experiments normalized as the % of unstimulated cells. ns, non significant. A single, double, triple, and four asterisks denote their significance of a p-value ≤ 0.05, ≤ 0.01, ≤ 0.001 and ≤ 0.0001 respectively in all experiments.

Upon TCR triggering, WASp regulates actin dynamics at the immune synapse by recruiting and activating the ARP2/3 complex (23). Thus, we verified if either ARP2/3 activity or actin polymerization is required for TCR-promoted inhibition of DGKα activity by using the ARP2/3 inhibitor CK666 or the actin polymerization inducer jasplakinolide. Jurkat cells were pre-treated for 30 minutes with either CK666 (60 μM) or jasplakinolide (0.5 μg/ml) before stimulation with anti-CD3 (UCHT1 10 μg/ml, 15 minutes) and quantification of DGKα enzymatic activity. As expected, DGKα activity was reduced by 50% upon TCR stimulation with no change in DGKα protein. Conversely, treatment with ARP2/3 inhibitor had a very mild effect on basal or TCR-inhibited DGKα activity and jasplakinolide showed no effect on basal condition (**Figures 2A, B**). As a readout of DAG signalling, we also assessed the phosphorylation of ERK1/2 using western blot analysis. Both the ARP2/3 inhibitor and jasplakinolide did not show any effects on ERK1/2 phosphorylation (**Figure 2C**), suggesting that ARP2/3 or actin polymerization does not participate in the control of DGKα and ERK1/2 activity. Overall, DGKα inhibition upon TCR stimulation is mediated by WASp independently from ARP2/3 activity. In the presence of SAP, this modulation of DGKα activity does not affect TCR signalling potency on the ERK1/2 pathway.

### 3.2 WASp binds and directly inhibits DGKα

**Figure 3:**
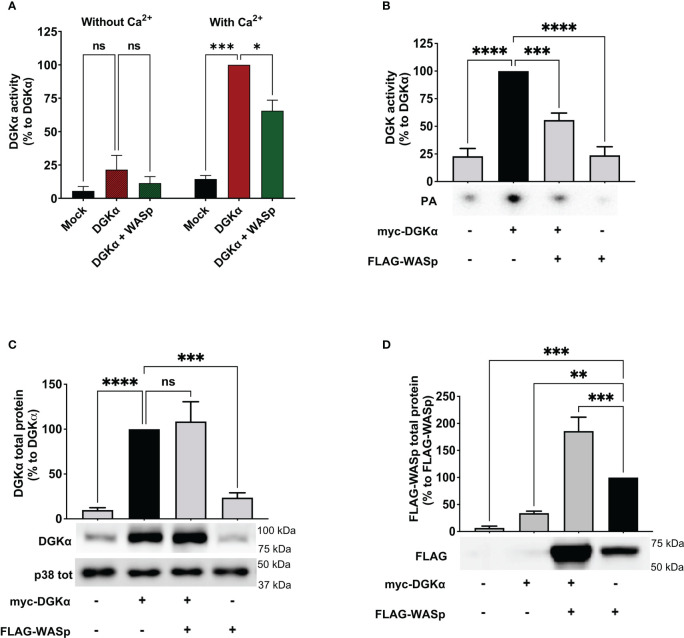
WASp directly inhibits DGKα activity **(A)** The activity of GST-DGKα was measured using the ADP-Glo™ assay (Promega) in the presence of an excess of WASp protein in the presence or absence of Ca^2+^. All the data have been normalized to DGKα with Ca^2+^ and are shown as the mean ± SEM of four independent experiments. **(B)** myc-DGKα and FLAG-WASp were transfected together or with GFP control vector in 293T-cells. 48 hours post-transfection, cells were collected, homogenized, and used in DGK activity assays normalized considering DGKα overexpressed homogenates as control; the lower panel is a representative assay and the upper panel is the mean ± SEM of five independent experiments. **(C)** Quantifications of myc-DGKα total protein as % of myc-DGKα transfected cells; the lower panel is a representative assay and upper panel is the mean ± SEM of five independent experiments. **(D)** Quantifications of FLAG-WASp total protein as % of FLAG-WASp transfected cells; the lower panel is a representative assay and upper panel is the mean ± SEM of five independent experiments. ns, non significant. A single, double, triple, and four asterisks denote their significance of a p-value ≤ 0.05, ≤ 0.01, ≤ 0.001 and ≤ 0.0001 respectively in all experiments.

Based on the involvement of WASp in the TCR-driven signalling cascade promoting DGKα inhibition, we verified whether WASp could bind DGKα and promote its inhibition. To verify this possibility, we measured the enzymatic activity of purified GST-DGKα protein in presence of an excess (1.5 – 3 fold) purified GST-WASp using a luciferase-based activity assay. As expected, DGKα is activated in the presence of Ca^2+^ and in this condition WASp partially inhibits DGKα activity (**Figure 3A**), indicating a direct inhibitory activity of WASp on DGKα.

To confirm if this inhibitory activity occurs in the cellular environment, we co-transfected myc-DGKα with either FLAG-WASp or GFP control vector in 293T cells and measured DGKα activity at 48 hours using total cell homogenates. In this system, DGKα is overexpressed and the DGK activity was proportional to the amount of transfected DGKα and was distinguishable from the endogenous background (**Figure 3B**). Notably, upon co-transfection with FLAG-WASp, the DGKα enzymatic activity was reduced by around 50% without any reduction of DGKα protein (**Figures 3B, C**). This observation indicates that WASp overexpression in 293T cells is sufficient to inhibit the DGKα activity without affecting its expression. We also noted that co-transfection with DGKα but not GFP control vector strongly enhances WASp protein levels (**Figure 3**), suggesting that the presence of DGKα promotes WASp stabilization similarly to what was previously reported for the WIP (29).

WASp is a hub for protein-protein interactions and active WASp is known to form a complex with CDC42 (20). Thus, we explored the possibility of DGKα regulation by this small GTPase. To verify this possibility, we co-transfected myc-DGKα with the constitutively active myc-CDC42 Q61L. Differently from what was observed upon co-transfection with FLAG-WASp, the presence of active CDC42 did not affect DGKα enzymatic activity or protein level in WASp absence reinforcing its central role in the control of DGKα activity (**Supplementary Materials 3**).

**Figure 4:**
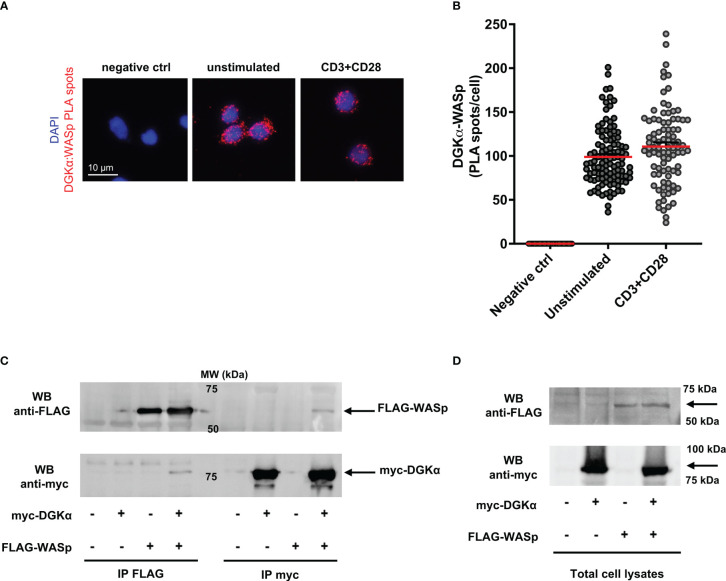
DGKα and WASp associate in a complex **(A)** Isolated human PBLs were seeded on poly-L-lysine coated glasses and stimulated for 15 minutes with anti-CD3 (UCHT1 10 μg/ml) and anti-CD28 (CD28.2, 5 μg/ml) antibodies, fixed, and permeabilized. PLA was performed to assess DGKα and WASp proximity; representative images are shown. **(B)** Quantification of the spots/cell resulting from PLA of two independent experiments are shown with mean ± SEM (n = 60 negative control; n = 109 unstimulated; n = 90 CD3+CD28). **(C)** Myc-DGKα and FLAG-WASp were co-expressed in 293T cells. After 48 hours, lysates were immunoprecipitated with either anti-FLAG (left side) or anti-myc (right side) antibodies. Immunoprecipitated proteins were analysed by western blotting with anti-FLAG antibody (upper panel) followed by anti-myc antibody after stripping (lower panel). The bands corresponding to FLAG-WASp and myc-DGKα are highlighted by black arrows. A representative experiment is shown out of three. **(D)** Total cell lysates from experiments shown in **(C)**.

Since we observed a role for WASp in controlling DGKα activity, we set to investigate whether the two proteins may associate. To verify if the endogenous proteins are in close proximity in primary T cells, we performed a Proximity Ligation Assay (PLA). Several fluorescent spots were detected in both unstimulated and anti-CD3/CD28 stimulated cells; in contrast, no signals were generated where primary antibodies were missing (negative control) (**Figure 4A**). Quantification of PLA events highlights the close association between DGKα and WASp, without a significant modulation driven by CD3-CD28 co-stimulation (**Figure 4B**).

To confirm the formation of a complex between DGKα and WASp, we co-expressed both the proteins in 293T cells and immunoprecipitated myc-DGKα and/or FLAG-WASp. The co-precipitation of the partner protein was verified by western blotting. We observed the presence of myc-DGKα in anti-FLAG immunoprecipitates when the two proteins were co-expressed, indicating the existence of a DGKα-WASp complex (**Figure 4C** left side). We also detected FLAG-WASp protein in the corresponding anti-myc immunoprecipitated lysate as a further confirmation of this interaction (**Figure 4C** right side). The input bands indicating equal transfection efficiency are shown in **Figure 4D**.

**Figure 5:**
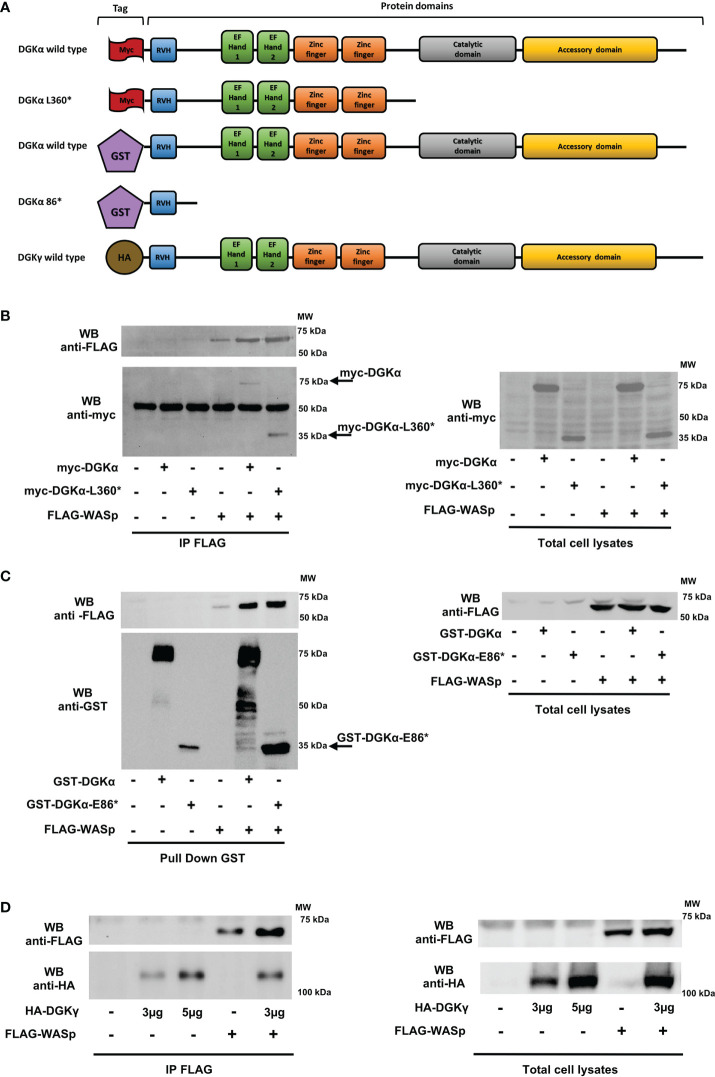
DGKα binds to WASp through the recoverin homology domain HEK 293-T cells were co-transfected with the indicated plasmids. 48 hours later, proteins were immunoprecipitated with anti-FLAG (**B** and **D**) or anti-GST antibody **(C)** and subsequently analysed by western blotting. **(A)** DGKα and DGKγ constructs used with domains and tags evidenced. **(B)** Interaction between FLAG-WASp and myc-DGKα WT or myc-DGKα-L360* deletion mutant and the corresponding total cell lysates. **(C)** Interaction between FLAG-WASp and GST-DGKα or GST-DGKα-E86* deletion mutant and the corresponding total cell lysates. **(D)** Interaction between FLAG-WASp and HA-DGKγ and the corresponding total cell lysates.

Moreover, to explore this DGKα-WASp complex formation and to identify their protein binding site, we performed the same immunoprecipitation assays using a series of DGKα and WASp deletion mutants. Initially, we used the myc-DGKα-L360* mutant, which lacks the C-terminal catalytic region (30). Our results (**Figure 5B**) demonstrate that this DGKα mutant retains the ability to bind WASp, indicating that WASp-DGKα interaction is mediated by the N-terminal DGKα regulatory domain. Thus, we produced the GST-DGKα E86* mutant maintaining the sole recoverin homology domain of DGKα; GST tag was used for this mutant as the corresponding myc tagged version is not sufficiently stable putatively due to the small size. As expected, glutathione beads pulled down GST-DGKα wt and the associated FLAG-WASp, confirming the interaction between the two. The observation that the GST-DGKα E86* mutant also pulled down FLAG-WASp indicates a role of the N-terminal recoverin homology domain of DGKα in this complex (**Figure 5C**). To further verify the specificity of this interaction, we repeated the co-immunoprecipitation assay with the highly homologous HA-DGKγ. We observed that the amount of HA-DGKγ immunoprecipitated by anti-FLAG antibodies depends on its overexpression but not by the presence of FLAG-WASp. Those data suggest that this isoform does not associate with FLAG-WASp (**Figure 5D**).

**Figure 6:**
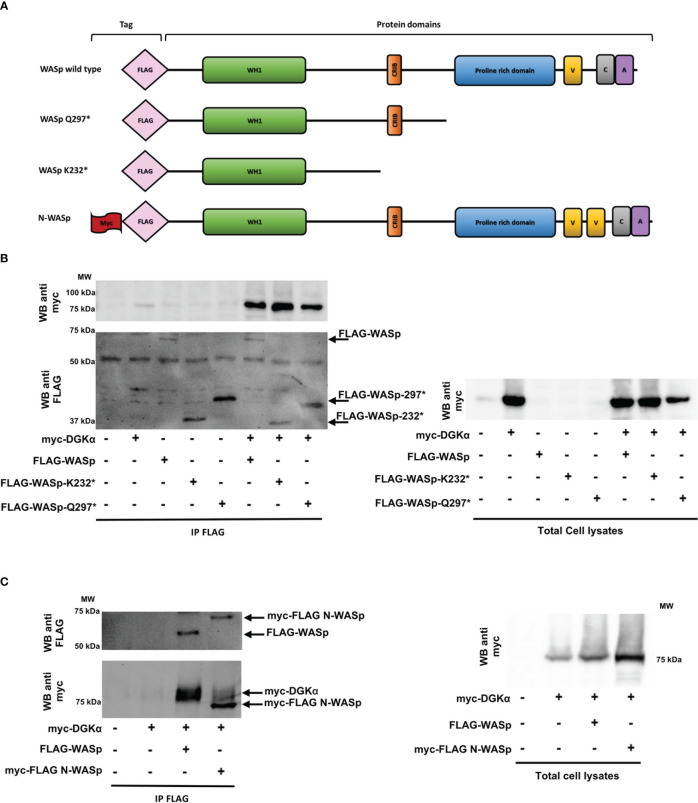
WASp interacts with DGKα through the WH1 domain while N-WASp does not bind HEK 293-T cells were co-transfected with the indicated plasmids. 48 hours later, proteins were immunoprecipitated with anti-FLAG antibody and subsequently analysed by western blotting.**(A)** WASP constructs used with domains and tags evidenced. **(B)** Interaction between myc-DGKα and FLAG-WASp or FLAG-WASp deletion mutants (FLAG-WASp K232* and FLAG-WASp Q297*) and the corresponding total cell lysates. **(C)** Interaction between FLAG-WASp or myc-FLAG N-WASp with myc-DGKα WT and the corresponding total cell lysates.

Conversely, we produced the FLAG-tagged WASp Q297* mutant lacking the C-terminal proline-rich and VCA domains and the WASp K232* mutant retaining the sole WH1 domain (**Figure 6A**). The observation that similar amounts of myc-DGKα wt co-immunoprecipitated with FLAG-WASp wt and the two deletion mutants indicate the role of the WH1 domain in the formation of the complex with DGKα (**Figure 6B**).

Lastly, to investigate the specificity of this interaction, we tested the interaction between myc-DGKα and myc-FLAG-N-WASp, a closely related WASp homologue featuring non-redundant functions. The immunoprecipitation with the anti-FLAG antibody revealed that myc-DGKα coimmunoprecipitates specifically with FLAG-WASp but not with myc-FLAG-N-WASp even if the latter is far more expressed (**Figure 6C**). This indicates a selectivity for WASp in the interaction with DGKα.

Taken together, these data demonstrate that DGKα and WASp can associate selectively by forming a stable complex mediated by WASp WH1 domain and DGKα recoverin homology domain. WASp presence inhibits DGKα enzymatic activity promoted by calcium, putatively impairing the shift to the active conformation for which the recoverin homology domain is required (31).

### 3.3 DGKα silencing or inhibition rescues IL-2 expression in WASp deficient T cells

DGKα inhibition is known to enhance TCR-promoted induction of IL-2 (32). Moreover, the lack of DGKα inhibition in SAP-deficient cells drives defective cytokine induction and RICD, which are rescued by the DGKα inhibition (14, 15). Since a similar defect in RICD and IL-2 induction is present in murine WASp deficient cells (22, 33), we evaluated the effect of DGKα inhibition on RICD, TCR signalling potency, and IL-2 production in WASp-deficient human lymphocytes. We silenced WASp in activated primary peripheral blood T lymphocytes (PBLs) using specific siRNA. To assess WASp silencing, WASp mRNA or protein levels were evaluated in all the experiments. At 4 days’ post silencing, we observed an 80% reduction of WASp mRNA and 70% decrease in WASp protein compared to control siRNA-transfected cells (**Supplementary Materials 4**).

**Figure 7:**
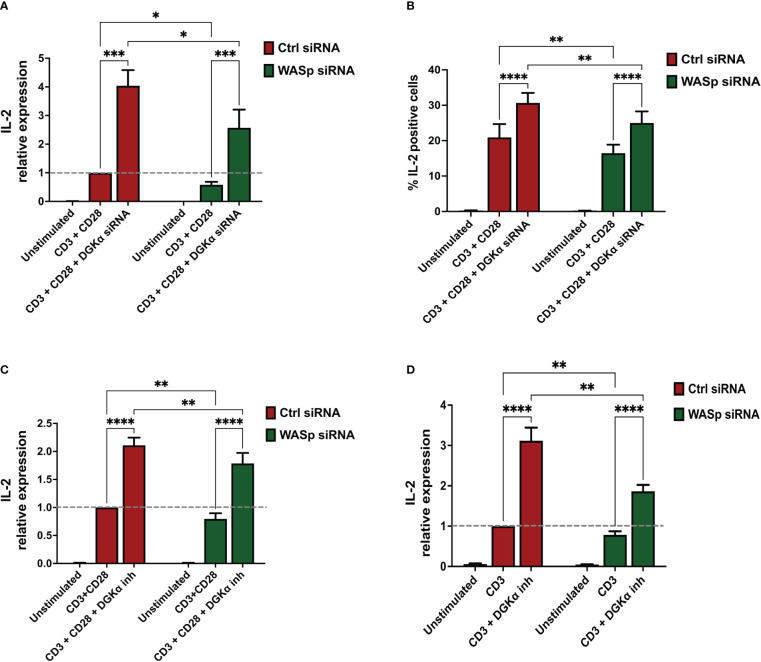
DGKα silencing or pharmacological inhibition rescues IL-2 defects in WASp deficient lymphocytes **(A)** Activated lymphocytes from healthy donors were transfected using siRNAs ctrl, WASp, and DGKα as indicated. After 4 days, transfected cells were restimulated using both OKT3 (1 µg/ml) and CD28.2 (1 µg/ml) for 4 hours followed by quantitative rt-PCR gene expression analysis to verify IL-2 expression. Data are the mean of five experiments from five individual healthy donors. **(B)** Activated lymphocytes from healthy donors were transfected using siRNAs ctrl, WASp, and DGKα as indicated. After 4 days, cells were seeded in RPMI 10% FBS and restimulated for 6 hours on immobilized anti-CD3 + anti-CD28 (clone UCHT1, 10 µg/ml and clone CD28.2 5 µg/ml, respectively). After 1 hour of incubation, brefeldin A (5 µg/ml) was added for 5 hours. Cells were permeabilized, stained with APC anti-human IL-2 antibody, and positive cells quantified by flow cytometry. Histogram shows the mean of the percentage IL-2 positive cells ± SEM of four independent replicates. **(C)** Activated lymphocytes from healthy donors were transfected using the indicated siRNA’s and after 4 days cells were restimulated with CD3 (clone OKT3 1 µg/ml, 4 hours) and CD28 (clone CD28.2, 1µg/ml, 4 hours) in the presence or absence of DGKα inhibitor (AMB639752 – 10 µM) **(D)** Activated lymphocytes from healthy donors were transfected using the indicated siRNA’s and after 4 days cells were restimulated with CD3 (clone OKT3 10 µg/ml, 4 hours) in the presence or absence of DGKα inhibitor (AMB639752 – 10 µM). Following this, IL-2 expression was evaluated by quantitative rt-PCR. Data are the mean ±SEM of five experiments from four individual healthy donors. A single, double, triple, and four asterisks denote their significance of a p-value ≤ 0.05, ≤ 0.01, ≤ 0.001 and ≤ 0.0001 respectively in all experiments.

To evaluate the effect on RICD, we re-stimulated control, WASp-, and SAP-silenced cells with increasing concentrations of anti-CD3 antibody (OKT3 from 0 to 100 ng/ml for 24 hours). We pre-treated all the cells with either vehicle (DMSO) or the DGKα-specific inhibitor AMB639752 (10 µM, 30 minutes). In line with our previous work (34), SAP deficiency reduced RICD, and this defect was partially compensated by AMB639752 treatment, which conversely did not affect RICD in control cells. In those experimental conditions, human PBLs silenced for WASp featured a normal RICD (**Supplementary Materials 5A**). In WASp-silenced cells we also evaluated TCR signalling outputs co-stimulating CD3 and CD28, notably NUR77 (NR4A1), FASL (FASLG), and FOS, which were robustly induced but were not affected by either WASp silencing or AMB639752 treatment (**Supplementary Materials 5**). In line with previous results on DAG signalling, those data suggest that WASp is less relevant for TCR signalling potency and RICD onset than SAP and that loss of the WASp inhibitory signalling on DGKα is not sufficient for the onset of apoptosis resistance. A different picture emerges when measuring IL-2 both at mRNA and protein levels in the same setting. In accordance with previous reports (11, 32), DGKα silencing or inhibition potentiated IL-2 production upon co-stimulation of CD3 and CD28 of control cells indicating the importance of DGKα enzymatic activity (**Figure 7**). siRNA-mediated WASp silencing resulted in a significant decrease in IL-2 mRNA and protein induced by TCR, indicating the key role of WASp in IL-2 production. We observed that both siRNA-mediated DGKα silencing or AMB639752 treatment restored IL-2 induction in WASP-silenced cells to levels higher than stimulated control cells, although not reaching control cells stimulated in the presence of DGKα inhibition or silencing (**Figure 7**). Those findings suggest the relevance of WASp-mediated DGKα activity inhibition in this signalling pathway. To verify if CD28 triggering is a requirement for this effect, we triggered the sole CD3 with higher doses (OKT3 10 μg/ml) and observed a similar potentiation of IL-2 expression by AMB639752 (10 μM) in control siRNA-transfected cells and a reduction in WASp-silenced cells which was rescued by the DGKα inhibitor (**Figure 7D**).

These observations imply that WASp-mediated inhibition of DGKα selectively regulates IL-2 gene expression and the % of IL-2 expressing cells without affecting the TCR signalling potency. In WASp absence DGKα is active, and its activity reduces IL-2 induction while TCR proapoptotic signalling is not influenced. This is different from what was observed with SAP silencing which reduces both IL-2 induction and RICD, suggesting that the WASp-DGKα axis is only a branch of the numerous SAP-dependent signalling pathways.

### 3.4 CDC42 and NCK-1 deficiencies reduce IL-2 expression, and this defect is rescued by DGKα inhibition

To characterize the role of SAP signalling in the control of IL-2 induction, we silenced selected SAP effectors and measured IL-2 induction.

**Figure 8:**
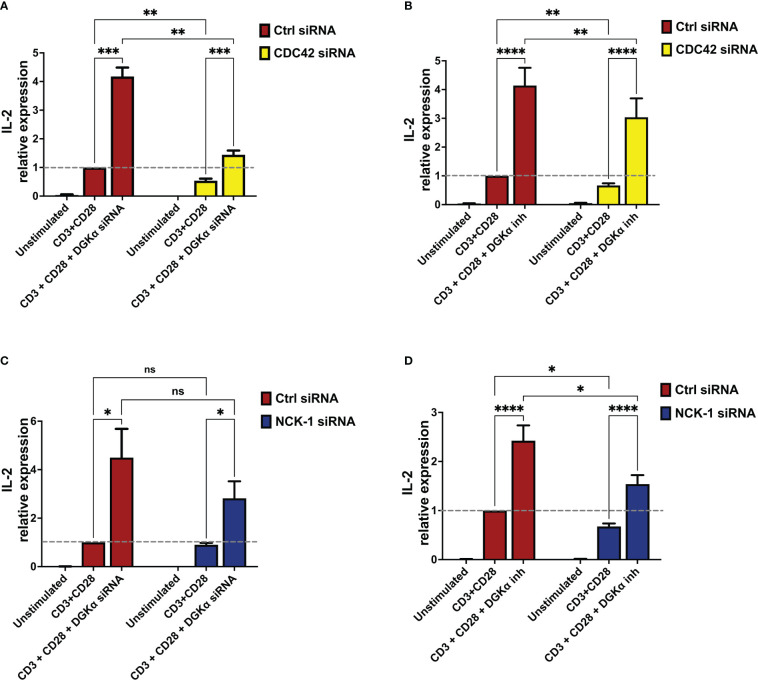
DGKα silencing or pharmacological inhibition rescues IL-2 defects in CDC42- and NCK-1-deficient lymphocytes **(A)** Activated lymphocytes from healthy donors were transfected using siRNAs ctrl, CDC42, and DGKα as indicated. After 4 days, transfected cells were restimulated using anti-CD3 (OKT3, 1 µg/ml) and anti-CD28 (CD28.2, 1 µg/ml) for 4 hours followed by quantitative rt-PCR gene expression analysis to verify IL-2 expression. Data are the mean ±SEM of six experiments from three individual healthy donors. **(B)** Activated lymphocytes from healthy donors were transfected using siRNAs ctrl or CDC42. After 4 days, transfected cells were restimulated using anti-CD3 (OKT3, 1 µg/ml) and anti-CD28 (CD28.2, 1 µg/ml) for 4 hours in the presence or absence of the DGKα specific inhibitor (AMB639752 - 10 µM), followed by quantitative rt-PCR gene expression analysis to verify IL-2 expression. Data are the mean ±SEM of eight experiments performed in triplicate from six individual healthy donors. **(C)** Activated lymphocytes from healthy donors were transfected using siRNAs ctrl, NCK-1, and DGKα as indicated. After 4 days, transfected cells were restimulated with anti-CD3 (OKT3, 1 µg/ml) and anti-CD28 (CD28.2, 1 µg/ml) for 4 hours followed by quantitative rt-PCR gene expression analysis to verify IL-2 expression. Data are the mean ±SEM of six experiments from four individual healthy donors. **(D)** Activated lymphocytes from healthy donors were transfected using siRNAs ctrl or NCK-1. After 4 days, transfected cells were restimulated using both anti-CD3 (OKT3, 1 µg/ml) and anti-CD28 (CD28.2, 1 µg/ml) for 4 hours in the presence or absence of the DGKα specific inhibitor (AMB639752 - 10 µM), followed by quantitative rt-PCR gene expression analysis to verify IL-2 expression. Data are the mean ±SEM of six experiments from six individual healthy donors for gene expression analysis and all experiments were performed in triplicate. ns, non significant. A single, double, triple, and four asterisks denote their significance of a p-value ≤ 0.05, ≤ 0.01, ≤ 0.001 and ≤ 0.0001 respectively in all experiments.

CDC42 is not only a key regulator of the WASp activity (20) but also a crucial SAP effector in the T cells (19). Thus, to verify its involvement in this signalling pathway, CDC42 was silenced in primary lymphocytes resulting in a 75% reduction of CDC42 mRNA 4 days after electroporation (**Supplementary Materials 6A, B, E**). After 4 days cells were restimulated, and quantitative rt-PCR revealed a consistent reduction of IL-2 induction by CD3 and CD28 co-stimulation in CDC42-silenced cells. When DGKα was silenced or inhibited in AMB639752 pre-treated cells, we observed potentiation of IL-2 induction in control cells and a rescue of defective IL-2 expression in CDC42-silenced cells, which is minor but reaches IL-2 levels of stimulated control cells (**Figures 8A, B**). This is in line with the major role of CDC42 as a WASp-DGKα pathway regulator.

NCK-1 is a binding partner for SAP and a direct regulator of TCR signalling along with the T cell proliferation (18). Interestingly, NCK-1 is also a WASp interactor, involved in WASp recruitment and activation at the TCR (21). Thus, we speculated that the NCK-1 adaptor could represent a crucial scaffold protein in this signalling pathway. To verify this hypothesis, we silenced NCK-1 expression in PBLs resulting in a 50% reduction of NCK-1 mRNA 4 days after electroporation (**Supplementary Materials 6C**, 6D and 6F). When we measured RICD induction and TCR-driven gene expression in the presence or absence of AMB639752 we observed a similar picture to what was obtained upon WASp silencing. We did not detect any alteration of either RICD, NUR77, or FASL induction in NCK-1 silenced cells co-stimulated with anti-CD3 and CD28 antibodies (**Supplementary Materials 7**). Also in this case, IL-2 induction was reduced upon NCK-1 silencing and rescued by DGKα inhibition to levels comparable of control stimulated cells but minor than control cells stimulated in the presence of DGKα knockdown (**Figure 8D**). A similar trend was observed with DGKα silencing: a reduction in IL-2 induction that was compensated by a strong induction upon DGKα silencing (**Figure 8C**). However, in co-silencing experiments, the reduction of IL-2 upon NCK-1 silencing failed to reach statistical significance; this may be due to the residual NCK-1 protein that may still play its function (**Supplementary Materials 6C, D, F**). Alternatively, other SAP effectors may participate in this pathway and compensate for NCK-1 absence.

Altogether these experiments indicate that CDC42 and NCK-1 contribute to the SAP signalling network controlling WASp and DGKα activity.

### 3.5 ARP2/3 activity is not required for IL-2 regulation by the WASp-DGKα complex

The CDC42-WASp signalling axis is a well-known regulator of actin branching polymerization through the ARP2/3 complex. To explore the role of the ARP2/3 complex in IL-2 induction by the TCR, PBLs were activated, cultured for 3 weeks, and pre-treated with ARP2/3 inhibitor CK666 at a concentration of 60 μM before restimulation (OKT3 1 μg/ml, CD28.2 1 μg/ml, 4 hours). When we measured IL-2 induction, we observed a normal induction by the TCR and potentiation by the DGKα inhibitor AMB639752 (10 μM). These results indicate that ARP2/3 is dispensable for the signalling pathway that controls IL-2 induction downstream to CDC42/WASp/DGKα (**Supplementary Materials 8**).

## 4 Discussion

**Figure 9:**
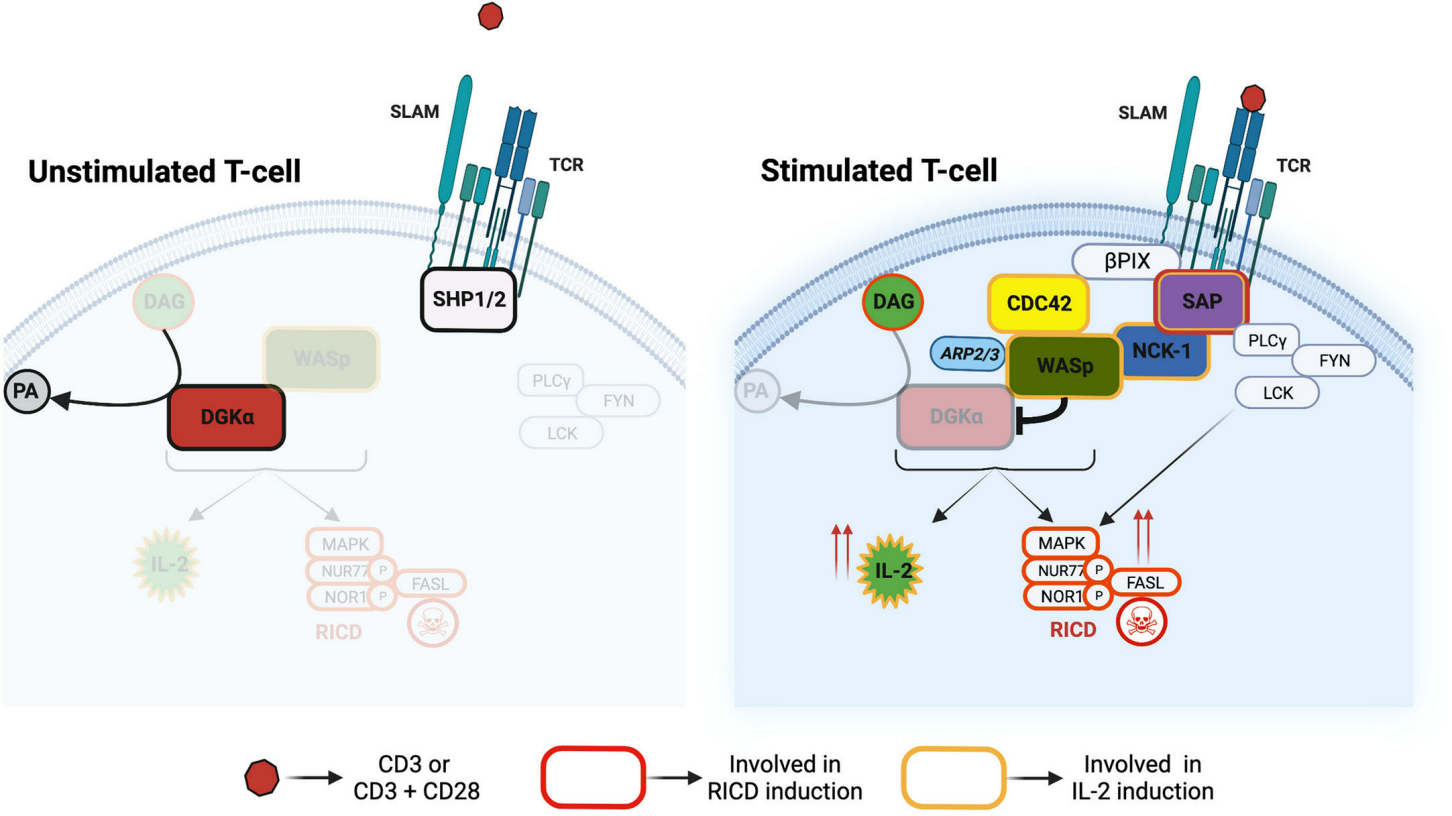
Divergent signalling pathways downstream to SAP The SAP-NCK-1/CDC42-WASp pathway controls DGKα activity and DAG cellular metabolism. This pathway tunes IL-2 and RICD responses in T cells. Other SAP interactors such as Lck and Fyn are crucial regulators of RICD onset.

In this present study, we explored the signalling pathways that mediate TCR-induced DGKα inhibition through the SAP adaptor protein. This pathway tunes cytokine induction and RICD in CD8^+^ cells (**Figure 9**), thus representing a target for the XLP-1 therapy (14).

We focused our attention on WASp as this protein is known to interact with molecules downstream to SAP such as CDC42 and NCK-1 (19, 35) and there are some similarities in the XLP-1 and WAS disease phenotypes (27). Our data demonstrate that DGKα and WASp are in close proximity in T cells and form a complex through the interaction between the DGKα recoverin homology domain with the WASp WH1 domain. This association is highly selective since it is not shared by the homologous DGKγ and N-WASp (**Figures 4**-**6**). The recoverin homology domain of DGKα is a short N-terminal domain that is involved in the maintenance of the autoinhibited conformation along with the two calcium-binding EF-hands (36). Indeed, those domains bind to the catalytic one inhibiting its activity and their removal results in a constitutively active, membrane-localized DGKα (31). The WASp WH1 domain is a protein-protein interaction domain able to bind the WIP chaperone and participate in WASp recruitment at the immune synapse (23, 29). Interestingly, we did not observe a modulation of PLA signal by CD3-CD28 co-stimulation, indicating that the two proteins are already in close proximity in non-stimulated cells (**Figure 4A**). The molecular details of this complex are unknown, but our findings suggest that a fraction of DGKα is associated to WASp, which constrain DGKα ability to respond to TCR-induced calcium waves. How this complex and its localization are modulated by signals such as CDC42 and NCK-1 acting on WASp or as Src tyrosine kinase acting on DGKα still awaits elucidation. Forcing DGKα/WASp complex formation by overexpression of both proteins inhibits DGKα (**Figure 3B**), while active CDC42 overexpression does not affect DGKα activity (**Supplementary Materials 3**). Similarly, *in vitro* coincubation of the purified proteins in the presence of calcium results in a direct inhibition of DGKα by WASp (**Figure 3A**). In line with this, WASp is required for DGKα inhibition by TCR triggering while ARP2/3 activity is dispensable (**Figures 1**,2). Those data suggest that DGKα is a novel direct WASp effector, and that the WASp function is independent of actin branching. Our results indicate that this interaction is specific for WASp, which is mainly expressed in hematopoietic cells, but not for its close homologue N-WASp. This observation could explain the peculiarity of T-cells where, upon TCR triggering, DGKα is inhibited. Conversely, in epithelial and endothelial cells, we and others observed DGKs’ activation upon membrane receptors triggering (30, 37, 38).

We observed that this WASp-mediated inhibition of DGKα activity is selectively important for IL-2 induction upon TCR stimulation at both mRNA and protein levels. Indeed, both SAP- and WASp-silenced cells feature a reduced IL-2 production which is rescued by DGKα silencing or inhibition to levels higher than stimulated control cells (**Figure 7** and (11, 25)). Differently from what was observed with SAP, WASp silencing in our experimental system does not alter the global profile of DAG species produced upon TCR triggering, nor does it affect the MAPK cascade, NUR77 and FASL induction, and RICD (**Supplementary Materials 2**, **4**). This suggests that the inhibition of DGKα by WASp affects small pools of DAG relevant specifically for IL-2 induction. The observation that the interaction is mediated by the WH1 domain of WASp, and that ARP2/3 activity appears unessential for IL-2 induction, reinforces the idea of WASp playing two separate functions in TCR signalling: actin branching morphogenesis on one side and modulation of DGKα activity on the other. This is in line with reports indicating that the defect of IL-2 synthesis in WASp-deficient cells is not due to decreased TCR signalling strength but to lack of ERK and nuclear factor of activated T cells’ (NF-AT) translocation into the nucleus and the consequent poor induction of the FOS transcription factor (25). However, in our experimental system, cFOS induction is nearly normal upon WASp silencing, indicating that this is not the major transcription factor involved in IL-2 induction upon DGKα silencing/inhibition (**Supplementary Materials 5**).

The absence of effect of DGKα silencing/inhibition on cells with normal RICD is in line with previous reports of a quantitatively minor role of DGKα compared to DGKζ in the T cell (6, 7). This minor role is indeed the effect of a TCR-driven inhibitory signal (2, 11) reducing DGKα activity at spatially selected DAG pools at the immune synapse (39). Indeed, we and others have observed that loss of DGKα activity in normal fully stimulated T cells has a very moderate effect on TCR signalling apart from a strong IL-2 induction and does not influence apoptosis sensitivity (14, 32). Conversely, previous studies from our lab and other groups evidenced that DGKα inhibition is an efficient way to restore T cell responses when the TCR signalling strength is reduced by anergy (40) or SAP absence in XLP-1 patients (14). A role for WASp in the control of RICD and autoimmunity was observed in lymphocytes from WASp KO mice (22) but not by us in human lymphocytes silenced for WASp; this discrepancy is putatively due to the experimental systems used (total genetic ablation in murine lymphocytes vs silencing in humans PBLs). Even if we have not explored this further, our data suggest a possible excessive DGKα activity in WAS patients that decreases the accumulation of selected DAG species. This could contribute to some of the defects in lymphocyte mobility and autoimmunity featured by WAS patients. It is interesting to note that DGKs also negatively regulate megakaryocytes’ maturation and platelets’ activation (41), suggesting that DGKα inhibitors may have a particularly positive effect on WAS patients.

In the effort to characterize the branch of SAP-mediated TCR signalling controlling DGKα activity, we observed that NCK-1 and CDC42 emerge among SAP effectors. Indeed, silencing CDC42 and, to a minor extent, NCK-1, decreases IL-2 induction in a DGKα-dependent manner (**Figures 8**, **9**). This shows that these two proteins act as putative participants to this signalling pathway as NCK-1 can bind both SAP and WASp with its SH3 domains (18, 20) while CDC42 is activated by SAP through βPIX exchange factor and is a major WASp regulator (19). However, the crucial DGKα regulator appears to be WASp as active CDC42 is not able to inhibit DGKα in the absence of WASp (**Supplementary Materials 3**) while WASp directly inhibits DGKα (**Figure 3A**).

Conversely, other SAP effectors, such as Lyn and Fyn, although important for TCR signalling and RICD, appear to be dispensable for the DGKα regulation (11) and justify the lack of full signalling rescue by the DGKα inhibition (14). Recent data also demonstrate that in SAP’s absence excessive SHP2 phosphatase activity contributes to XLP-1 phenotypes such as RICD (42). Those qualitative differences between SAP and WASp signalling render SAP more crucial for the control of RICD than WASp. However, our data indicate that the increase of DAG signalling due to pharmacological DGKα inhibition may compensate quantitatively for alterations of TCR signalosome due to SAP silencing. Intriguingly, SAP is a novel PD-1 signal transducer (43) suggesting that this pathway may also be involved in the control of DGKα activity of tumour infiltrating lymphocytes. This paves the way for the use of DGKα inhibitors not only in primary immunodeficiencies but also in other T cell hyporesponsive states such as tumour-induced anergy/exhaustion (44). Indeed, tumour infiltrating lymphocytes feature reduced WASp activation (45) while active WASp potentiates T cells and NK lytic function similarly to what was observed with DGKα inhibitors (4, 46).

WASp plays a primary role in the organization of actin dynamics at the immune synapse (24, 47). As the actin organization is not involved in the control of DGKα activity and IL-2 induction, we have not explored this issue further. However, the regulation of DGKα activity may represent an actin-independent function of WASp at the immune synapse as precise localization of DGKα activity is crucial for IS organization (8).

Concluding, we observed that DGKα inhibition is a novel function of WASp independent from ARP2/3 driven actin branching. This regulation allows WASp to tune IL-2 production upon TCR triggering. Those data suggest a potential utility of DGK inhibitors for WAS treatment.

## Data availability statement

The original contributions presented in the study are included in the article/supplementary material. Further inquiries can be directed to the corresponding author.

## Author contributions

Conceptualization and study design: GB, AG, SV, ER, and VM; Methodology and investigation: Biochemical assays - SV, SC, LR, and GB; Lipidomic experiments – BP and MMan; Biological assays - SV, SC, GR, SM, and VM; Formal analysis: SV, SC, GB, BP, and MMal; Resources: AA, MMan, and MMal; Writing - original draft: GB, SV, and SC; Writing - review and editing: GB, SV, SC, AA, BP, MMan, GR, VM, and AG; Funding acquisition: GB and AG; Supervision: GB, AG, and MMan; All authors read and approved the final version of the manuscript.

## Glossary

**Table:**

DGKα	diacylglycerol kinase alpha
DGKζ	diacylglycerol kinase zeta
DAG	Diacylglycerol
WASp	Wiskott-Aldrich syndrome protein
WAS	Wiskott-Aldrich syndrome
NPFs	nucleation-promoting factors
SHP-1	Src homology phosphatase 1
SHP-2	Src homology phosphatase 2
βPIX	Pak interacting exchange factor
TCR	T cell receptor
ARP2/3	actin-related protein 2/3
CDC42	cell division control protein 42
NCK-1	NCK adaptor protein 1
SAP	SLAM-associated protein
SLAM	signalling lymphocyte activation molecule
RICD	restimulation-induced cell death
XLP-1	X-linked lymphoproliferative disease 1
IL-2	interleukin 2
NF-AT	nuclear factor of activated T cells
ERK1/2	extracellular signal-regulated kinase 1 and 2
WIP	WASp interacting protein
FASL	FAS ligand
NUR77	nuclear receptor 4A1
FOS	Finkel-Biskis-Jinkis osteosarcoma gene
IFNγ	Interferon gamma
PBLs	peripheral blood lymphocytes
BFA	Brefeldin A
