# Supplementary material for: Wiskott-Aldrich syndrome protein interacts and inhibits diacylglycerol kinase alpha promoting IL-2 induction
Source: Front Immunol. 2023 Apr 17;14:1043603. doi: 10.3389/fimmu.2023.1043603 (PMC10149931; doi:10.3389/fimmu.2023.1043603)
Supplement: Supplementary file 1 [file DataSheet_1.docx]

Supplementary Material

# Supplementary Figures and Tables

**Table S1.** Complete list of identified DAG species in unstimulated and stimulated (CD3+CD28 1µg/ml) Jurkat cells.

| **DAG Species** | **Mean Unstimulated Jurkat Cells** | **Standard Deviation Unstimulated Jurkat Cells** | **Mean OKT3+CD28.2 Jurkat Cells** | **Standard Deviation OKT3+CD28.2 Jurkat Cells** | ***p*-value** |
| --- | --- | --- | --- | --- | --- |
| DAG 17:0 | 0.2506 | 0.1637 | 0.2564 | 0.1863 | ns |
| DAG 22:1 | 0.2636 | 0.1355 | 0.2670 | 0.1356 | ns |
| DAG 23:1 | 0.6520 | 0.1573 | 0.5636 | 0.2247 | ns |
| DAG 24:3 | 0.4778 | 0.0681 | 0.4502 | 0.0629 | ns |
| DAG 25:0 | 0.7549 | 0.0321 | 0.7172 | 0.0691 | ns |
| DAG 28:2 | 0.6569 | 0.0765 | 0.6674 | 0.0897 | ns |
| DAG 30:5 | 0.6681 | 0.0983 | 0.6686 | 0.0870 | ns |
| DAG 30:6 | 0.8293 | 0.0529 | 0.8384 | 0.1483 | ns |
| DAG 32:0 | 0.7085 | 0.0990 | 0.7552 | 0.0174 | ns |
| DAG 34:0 | 0.3154 | 0.0376 | 0.3412 | 0.0477 | ns |
| DAG 34:0\|DAG 16:0_18:0 | 0.2744 | 0.0214 | 0.3103 | 0.0462 | ns |
| DAG 34:1 | 0.5121 | 0.0566 | 0.6307 | 0.0385 | ns |
| DAG 34:1\|DAG 16:0_18:1 | 0.3850 | 0.0316 | 0.4439 | 0.0314 | ns |
| DAG 35:6 | 1.6901 | 1.8302 | 1.0136 | 0.6972 | ns |
| DAG 36:0 | 0.3309 | 0.0286 | 0.3165 | 0.0640 | ns |
| DAG 36:0\|DAG 16:0_20:0 | 0.2680 | 0.0399 | 0.2836 | 0.0540 | ns |
| DAG 36:1 | 0.3165 | 0.0275 | 0.4229 | 0.0443 | ns |
| DAG 36:1\|DAG 16:0_20:1 | 0.3605 | 0.0242 | 0.4429 | 0.0571 | ns |
| DAG 36:2 | 0.5293 | 0.0733 | 0.6624 | 0.0501 | ns |
| DAG 36:2\|DAG 18:1_18:1 | 0.4000 | 0.0624 | 0.4876 | 0.0530 | ns |
| DAG 36:3\|DAG 18:1_18:2 | 0.7575 | 0.3573 | 0.6810 | 0.0862 | ns |
| DAG 37:6 | 0.5554 | 0.4140 | 0.4703 | 0.2196 | ns |
| DAG 37:7 | 0.8551 | 0.0991 | 0.9722 | 0.3120 | ns |
| DAG 38:1 | 0.3401 | 0.0239 | 0.2931 | 0.0346 | ns |
| DAG 38:2 | 0.3791 | 0.0518 | 0.5707 | 0.0729 | 0.0052 |
| DAG 38:2\|DAG 18:1_20:1 | 0.3646 | 0.0245 | 0.5170 | 0.0628 | 0.004 |
| DAG 38:3 | 0.4272 | 0.0865 | 0.8245 | 0.0680 | <0.0001 |
| DAG 38:3\|DAG 18:0_20:3 | 0.3675 | 0.0420 | 0.7062 | 0.0731 | 0.0002 |
| DAG 38:3\|DAG 18:1_20:2 | 0.3691 | 0.0415 | 0.7092 | 0.0723 | 0.0002 |
| DAG 38:4\|DAG 18:1_20:3 | 0.5484 | 0.0412 | 0.8564 | 0.1110 | 0.002 |
| DAG 39:6 | 0.5496 | 0.4767 | 0.4447 | 0.2569 | ns |
| DAG 40:1\|DAG 16:0_24:1 | 0.4538 | 0.0608 | 0.4866 | 0.0310 | ns |
| DAG 40:2 | 0.8784 | 0.0098 | 0.8898 | 0.0679 | ns |
| DAG 40:2\|DAG 18:1_22:1 | 0.4118 | 0.0436 | 0.4038 | 0.0946 | ns |
| DAG 40:3 | 0.3330 | 0.0574 | 0.7750 | 0.1029 | 0.0003 |
| DAG 40:4\|DAG 18:0_22:4 | 0.4181 | 0.0671 | 0.6951 | 0.0728 | 0.0014 |
| DAG 40:5\|DAG 18:0_22:5 | 0.4854 | 0.0818 | 0.8125 | 0.1750 | 0.0147 |
| DAG 40:6 | 0.7557 | 0.1679 | 0.7490 | 0.2671 | ns |
| DAG 40:6\|DAG 16:0_24:6 | 1.181 | 0.0850 | 1.110 | 0.0913 | ns |
| DAG 41:6 | 0.5308 | 0.3610 | 0.4238 | 0.1756 | ns |
| DAG 41:6\|DAG 15:0_26:6 | 1.0663 | 0.1199 | 1.1133 | 0.1484 | ns |
| DAG 42:2\|DAG 18:1_24:1 | 0.4207 | 0.0451 | 0.4706 | 0.0424 | ns |
| DAG 42:6\|DAG 16:0_26:6 | 0.9391 | 0.0535 | 0.9725 | 0.0637 | ns |
| DAG 43:6\|DAG 17:0_26:6 | 0.8259 | 0.1823 | 0.9254 | 0.0669 | ns |
| DAG 44:2\|DAG 18:1_26:1 | 0.3992 | 0.0707 | 0.4618 | 0.0954 | ns |
| DAG 48:1\|DAG 16:0_32:1 | 0.4821 | 0.1278 | 0.4363 | 0.2395 | ns |
| DAG 48:2\|DAG 16:0_32:2 | 0.4470 | 0.0938 | 0.4677 | 0.0545 | ns |
| DAG 50:2\|DAG 16:1_34:1 | 0.5762 | 0.1115 | 0.4229 | 0.1130 | ns |
| DAG 50:6\|DAG 16:0_34:6 | 0.8114 | 0.0556 | 0.7617 | 0.0717 | ns |
| DAG 52:2\|DAG 16:0_36:2 | 0.3976 | 0.0823 | 0.3605 | 0.0965 | ns |
| DAG O-32:0\|  DAG O-16:0_16:0 | 0.7116 | 0.0942 | 0.6024 | 0.0505 | ns |
| DAG O-34:0\|  DAG O-18:0_16:0 | 0.1582 | 0.1103 | 0.1911 | 0.0330 | ns |
| DAG O-34:1\|  DAG O-18:1_16:0 | 0.3299 | 0.1074 | 0.3187 | 0.0460 | ns |
| DAG O-37:2\|  DAG O-21:1_16:1 | 1.016 | 0.1595 | 1.166 | 0.0540 | ns |


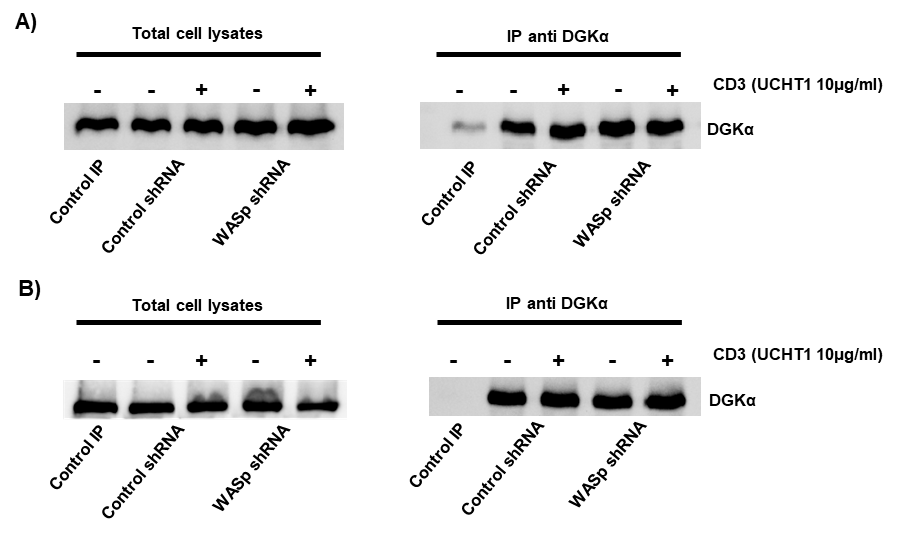


**Supp. Mat. 1: DGKα immunoprecipitation is not affected by stimulation in both control shRNA and WASp shRNA Jurkat cells.**

Control shRNA and WASp shRNA Jurkat cells (3 x 10^7^) were stimulated with CD3 agonist (UCHT1 10 μg/ml for 15 minutes). Post-stimulation, cells were lysed and total cell lysates were analysed by western blotting with anti DGKα antibodies (left panel) or immunoprecipitated with anti-DGKα antibody followed by western blotting with the same antibody (right panel). A and B indicates two independent experiments.


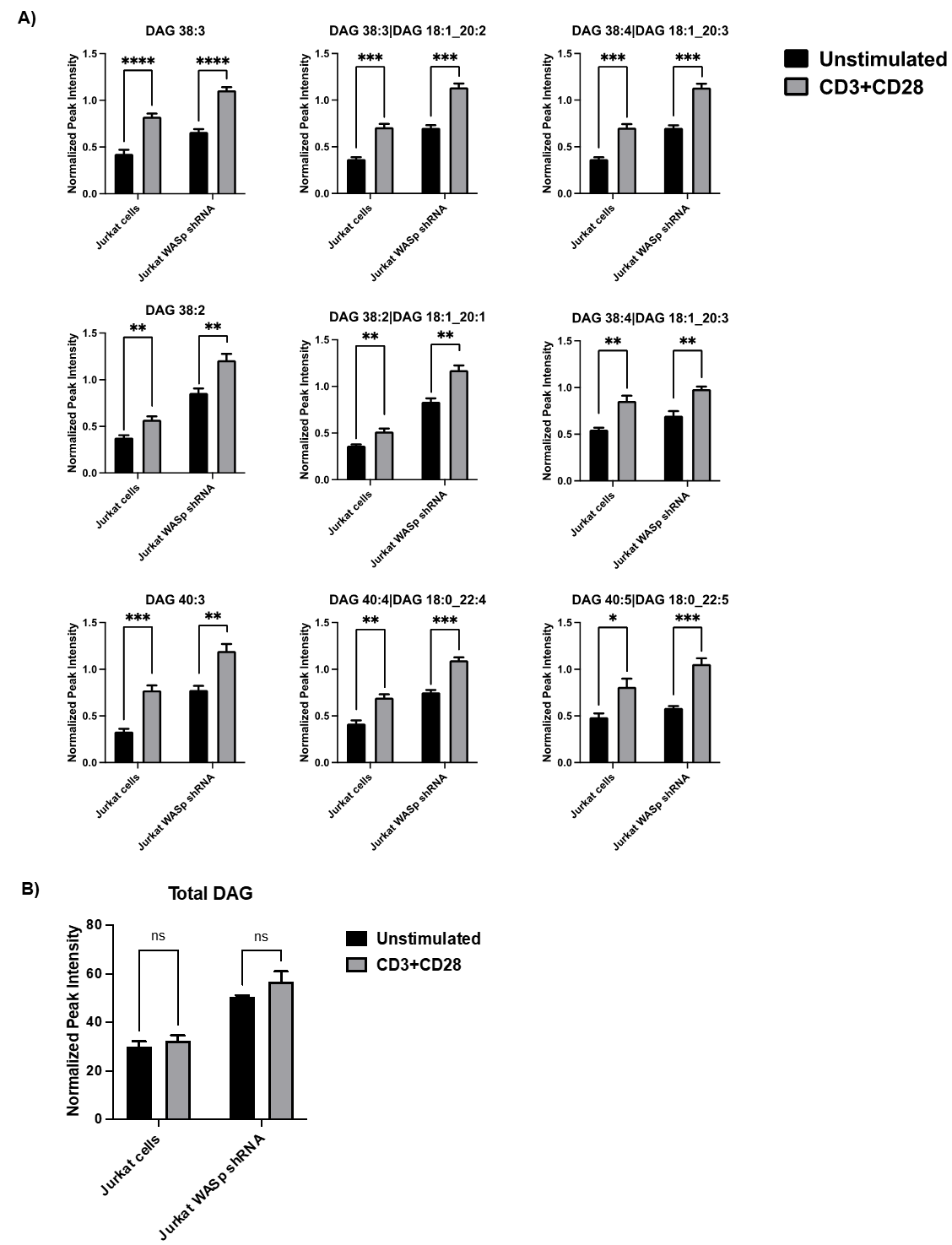


**Supp. Mat. 2: No significant differences were observed in DAG fluctuations between Jurkat cells and Jurkat WASp shRNA cells**

1. Single DAG species modulation associated with Jurkat cells unstimulated *vs* stimulated with OKT3 and CD28.2 and Jurkat WASp shRNA unstimulated *vs* stimulated with OKT3 and CD28.2.
2. Total DAG fluctuations in Jurkat cells unstimulated vs stimulated with OKT3 and CD28.2 and Jurkat WASp shRNA unstimulated *vs* stimulated with OKT3 and CD28.2.


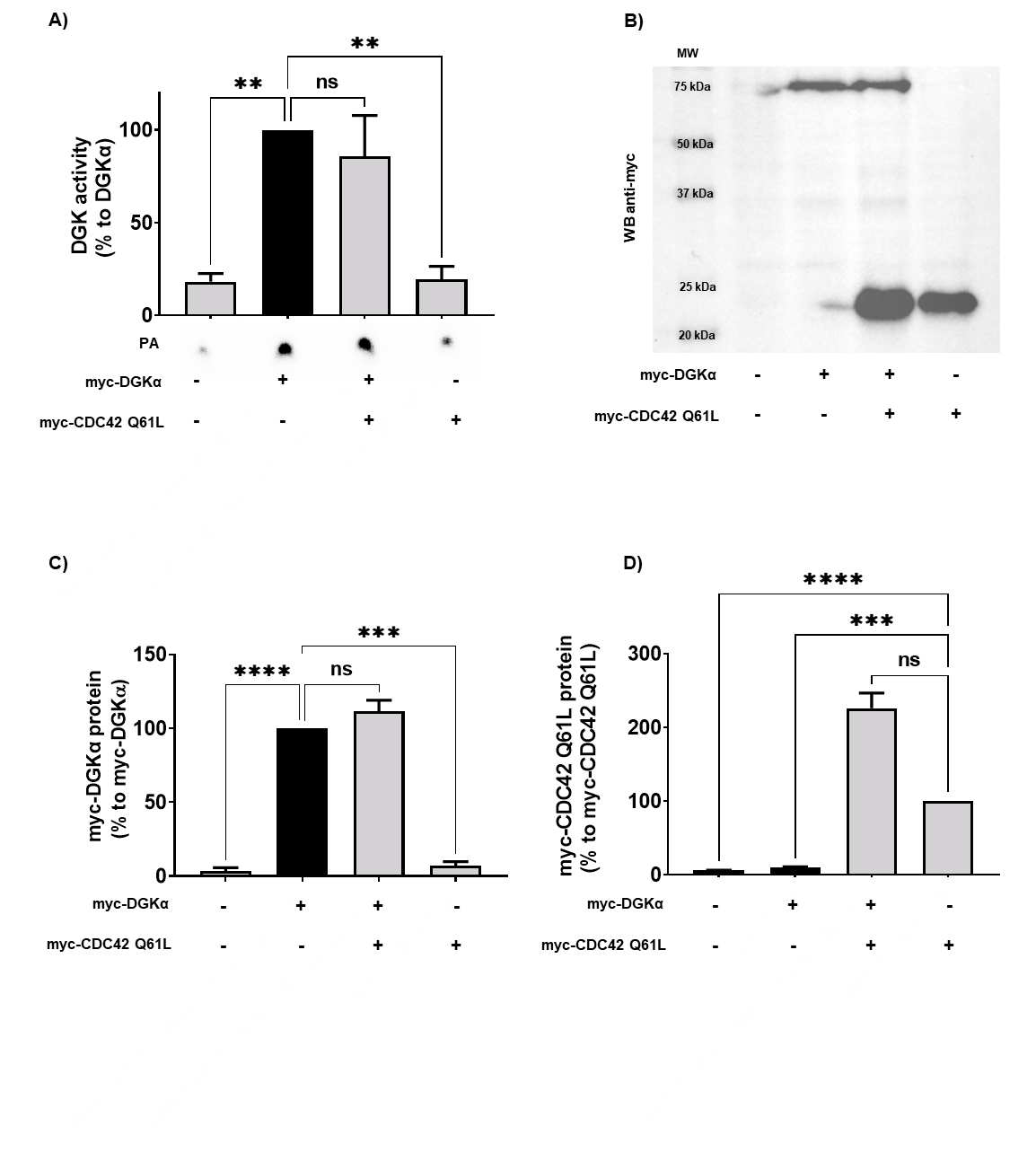


**Supp. Mat. 3: CDC42 Q61L overexpression does not inhibit DGKα**

Myc-DGKα and myc-CDC42 Q61L were transfected either alone or together in 293T-cells. 48h post-transfection, cells were collected, homogenized and used in:

1. DGKα activity assays. A representative experiment is shown together with the mean ± SEM of 4 independent experiments.
2. A representative western blot using anti-myc antibody.
3. Quantification of myc-DGKα in the corresponding homogenates normalised for myc-DGKα single transfection at 100%

Quantification of myc-CDC42 Q61L in the corresponding homogenates normalised for myc-CDC42 Q61L single transfection at 100%.


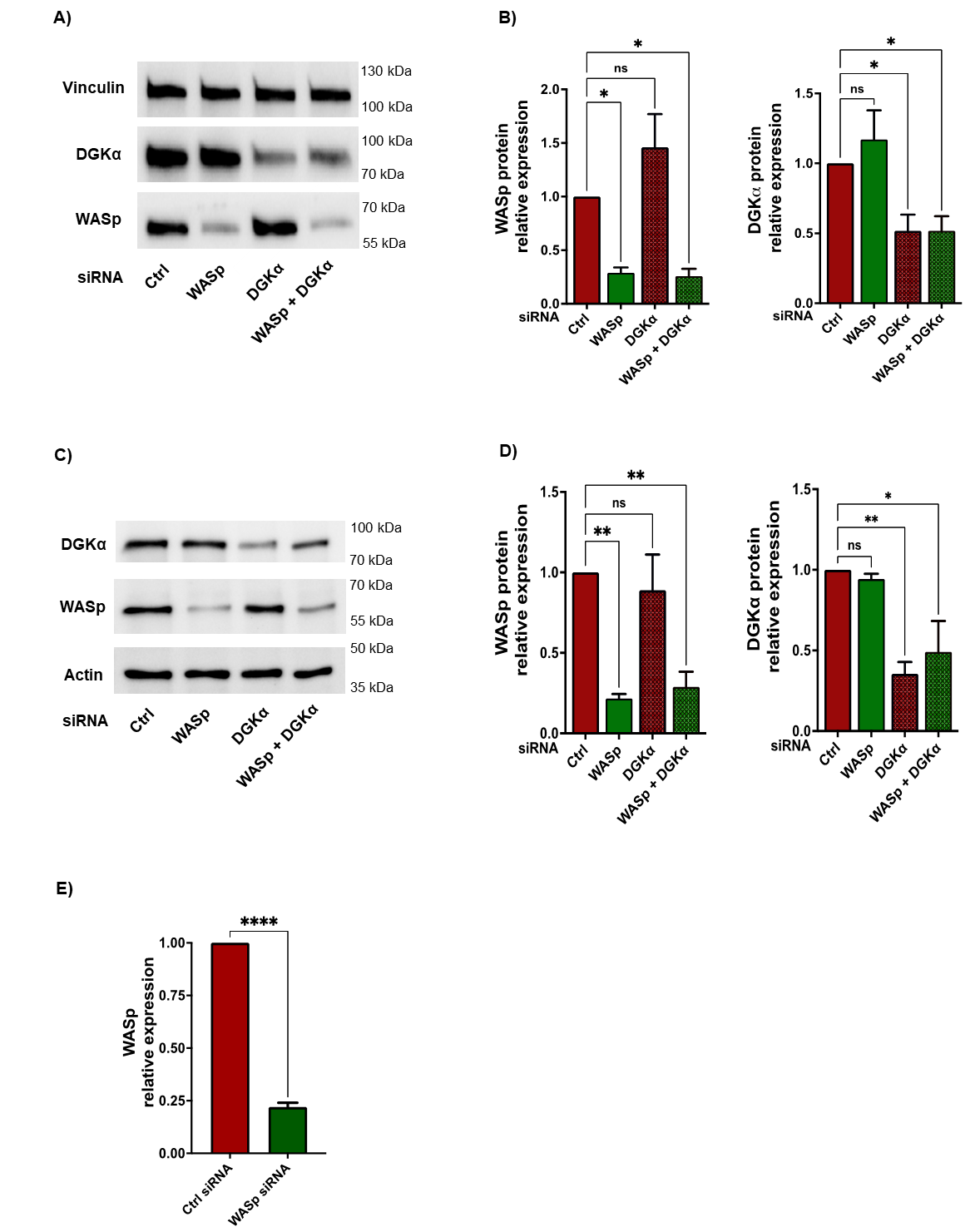


**Supp. Mat. 4: Silencing controls of Figure 7: DGKα silencing or pharmacological inhibition rescues IL-2 defects in WASp deficient lymphocytes**

1. WASp and DGKα expression in PBLs of a representative donor from Fig. 7A were evaluated by western blotting using vinculin as a normalizer.
2. WASp and DGKα protein quantification (relative to ctrl) related to the experimental data presented in A and Fig. 7A.
3. WASp and DGKα expression in a representative donor from Fig. 7B were evaluated by western blotting using actin as a normalizer.
4. WASp and DGKα protein quantification (relative to ctrl) related to the experimental data presented in C and Fig. 7B.
5. Quantitative rt-PCR gene expression analysis of WASp mRNA related to the experimental data presented in Fig. 7C and 7D.


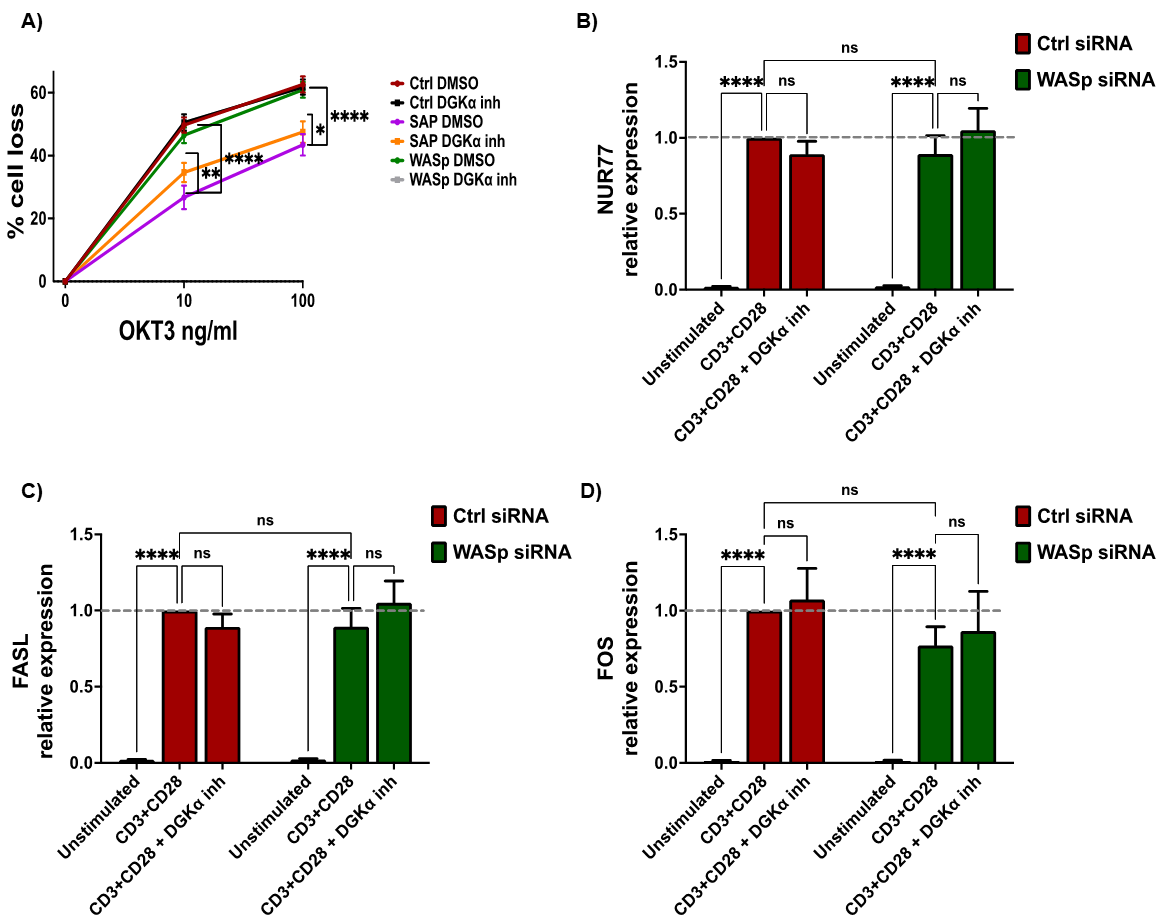


**Supp. Mat. 5: WASp deficiency neither effect RICD nor NUR77, FASLG or FOS expression**

Activated lymphocytes from healthy donors were transfected using the indicated siRNA’s and after 4 days cells were restimulated with:

- increasing concentrations of OKT3 (A) for 24 hrs either in the presence or absence of specific DGKα inhibitor (AMB639752 – 10 µM) and the % of cell loss was evaluated by FACS using PI staining to evaluate RICD. Data are the mean $\pm\mathrm{SEM}$ of 14 experiments from 9 individual healthy donors.
- OKT3 (1 µg/ml) and CD28.2 (1 µg/ml) for 4 hrs in the presence or absence of DGKα inhibitor (AMB639752 – 10 µM) followed by quantitative rt-PCR gene expression analysis to verify NUR77 (B), FASL (C) and FOS (D). Data are the mean $\pm\mathrm{SEM}$ of at least 4 experiments from 4 individual healthy donors.


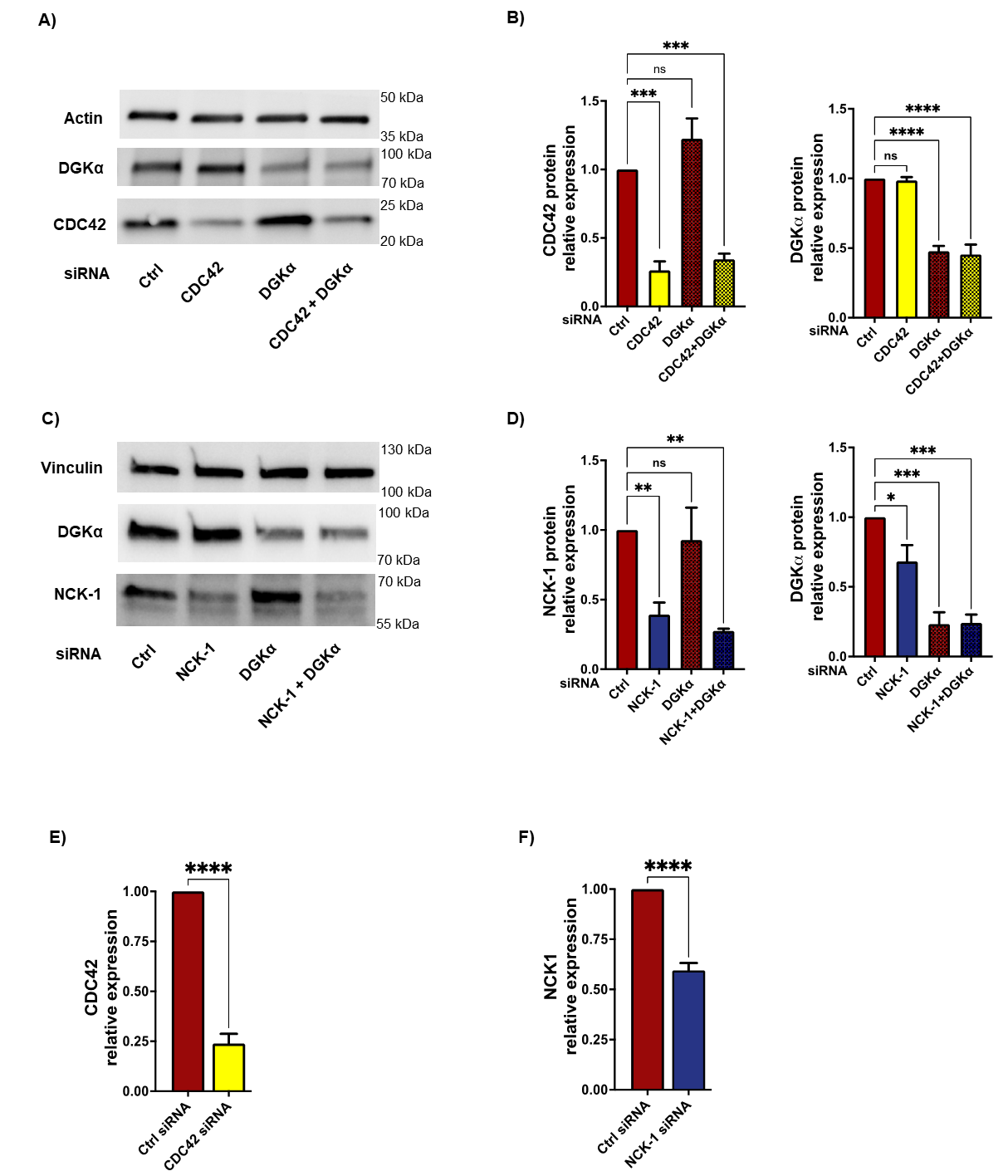


**Supp. Mat. 6: Silencing controls of Figure 8 DGKα silencing or pharmacological inhibition rescues IL-2 defects in CDC42 and NCK-1 deficient lymphocytes**

1. CDC42 and DGKα expression in PBLs of a representative donor from Fig. 8A were evaluated by western blotting using actin as a normalizer.
2. CDC42 and DGKα protein quantification (relative to ctrl) related to the experimental data presented in A and Fig. 8A.
3. NCK-1 and DGKα expression in a representative donor from Fig. 8C were evaluated by western blotting using vinculin as a normalizer.
4. NCK-1 and DGKα protein quantification (relative to ctrl) related to the experimental data presented in C and Fig. 8C.
5. Quantitative rt-PCR gene expression analysis of CDC42 mRNA related to the experimental data presented in Fig. 8B.
6. Quantitative rt-PCR gene expression analysis of NCK-1 mRNA related to the experimental data presented in Fig. 8D.


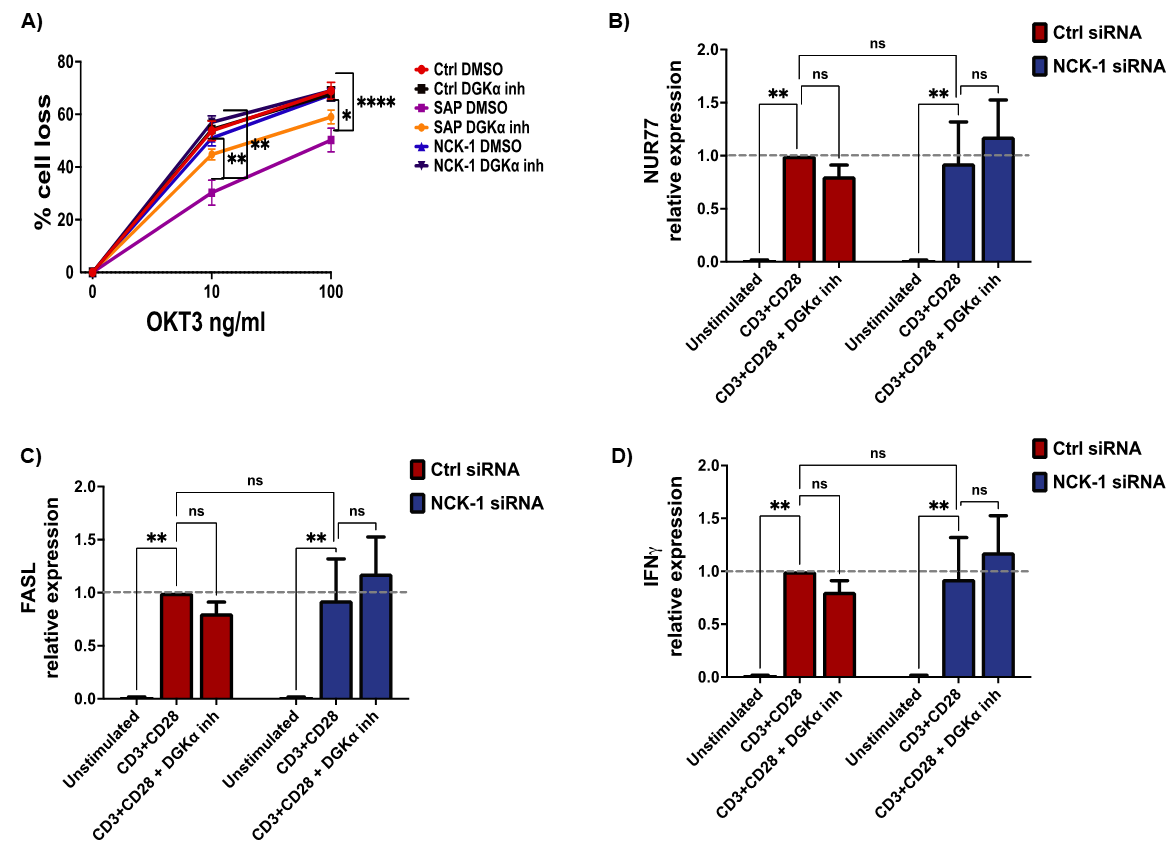


**Supp. Mat. 7: NCK-1 silencing neither effect RICD nor NUR77, FASLG or IFNγ expression**

Activated lymphocytes from healthy donors were transfected using the indicated siRNA’s and after 4 days cells were restimulated with:

- increasing concentrations of OKT3 (A) for 24 hours either in the presence or absence of specific DGKα inhibitor (AMB639752 – 10 µM) and the % of cell loss was evaluated by FACS using PI staining to evaluate RICD. Data are the mean $\pm\mathrm{SEM}$ of 4 experiments from 4 individual healthy donors.
- OKT3 (1 µg/ml) and CD28.2 (1 µg/ml) for 4 hours in the presence or absence of DGKα inhibitor (AMB639752 – 10 µM) followed by quantitative rt-PCR gene expression analysis to verify NUR77 (B), FASL (C) or IFNγ (D). Data are the mean $\pm\mathrm{SEM}$ of 2 experiments from 2 individual healthy donors.

**
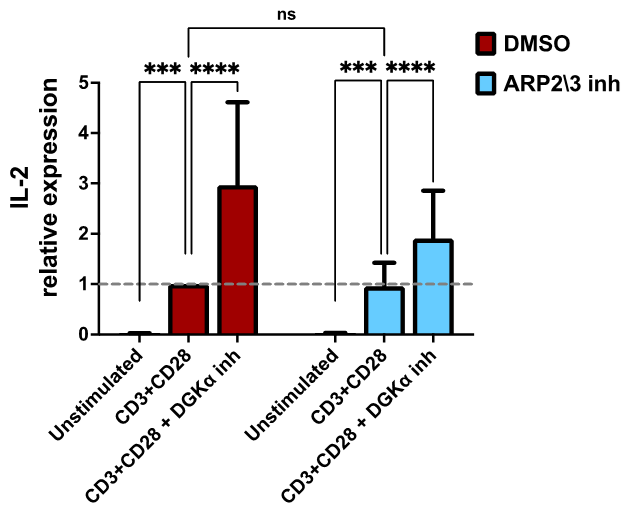
**

**Supp. Mat. 8: ARP2/3 inhibition has no effect on IL-2 expression**

Activated lymphocytes from healthy donors were treated with ARP2/3 inhibitor (CK666 – 60 µM) for 30 minutes and restimulated with OKT3 (1 µg/ml) and CD28.2 (1 µg/ml) for 4 hours in the presence or absence of DGKα inhibitor (AMB639752 – 10 µM) followed by quantitative rt-PCR gene expression analysis to verify IL-2 expression. Data are the mean $\pm\mathrm{SEM}$ of 9 experiments from 6 healthy donors.
